# Non‐genetic factors associated with ACE‐inhibitor and angiotensin receptor blocker‐induced angioedema

**DOI:** 10.1002/clt2.70058

**Published:** 2025-05-07

**Authors:** Diana Dubrall, Nora L. Branding, Carina M. Mathey, Anna M. Weber, Michael Steffens, Maike Below, Matthias Schmid, Bettina Wedi, Dorothea Wieczorek, Philipp M. Amann, Harald Löffler, Lukas Koch, Clemens Schöffl, Heinrich Dickel, Nomun Ganjuur, Thorsten Hornung, Timo Buhl, Emel Aygören‐Pürsün, Gerda Wurpts, Jens Greve, Markus M. Nöthen, Andreas J. Forstner, Bernhardt Sachs

**Affiliations:** ^1^ Institute for Medical Biometry Informatics and Epidemiology University Hospital of Bonn Bonn Germany; ^2^ Research Division Federal Institute of Drugs and Medical Devices (BfArM) Bonn Germany; ^3^ Institute of Human Genetics University of Bonn School of Medicine and University Hospital Bonn Bonn Germany; ^4^ Central Research Institute for Ambulatory Health Care in Germany Berlin Germany; ^5^ Klinik für Dermatologie Allergologie und Venerologie Medizinische Hochschule Hannover Hannover Germany; ^6^ Klinik für Dermatologie Allergologie und Phlebologie SLK‐Kliniken Heilbronn Heilbronn Germany; ^7^ Department of Medicine Faculty of Medicine and Dentistry Danube Private University Krems Austria; ^8^ Department of Dermatology and Venereology Medical University of Graz Graz Austria; ^9^ Department of Dermatology, Venereology and Allergology St. Josef Hospital University Medical Center Ruhr University Bochum Bochum Germany; ^10^ Zentrum für Hauterkrankungen Universitätsklinikum Bonn Bonn Germany; ^11^ Klinik für Dermatologie Venerologie und Allergologie Universitätsmedizin Göttingen Göttingen Germany; ^12^ Department of Pediatrics Goethe University Frankfurt Frankfurt Germany; ^13^ Klinik für Dermatologie und Allergologie ‐ Hautklinik Universitätsklinik der RWTH Aachen Aachen Germany; ^14^ Department of Otorhinolaryngology—Head and Neck Surgery Ulm University Medical Center Ulm Germany; ^15^ Institute of Neuroscience and Medicine (INM‐1) Research Center Jülich Jülich Germany; ^16^ Department for Dermatology and Allergy University Hospital RWTH Aachen Aachen Germany

**Keywords:** angioedema, angiotensin‐converting enzyme inhibitors, angiotensin‐receptor blockers, drug‐induced angioedema, spontaneous reports

## Abstract

**Background:**

Angioedema (AE) rarely occurs as a potentially life‐threatening adverse drug reaction (ADR) to angiotensin‐converting enzyme inhibitors (ACEi) or angiotensin receptor blockers (ARB). The aim of the present study was to investigate non‐genetic association factors with ACEi‐/ARB‐induced angioedema in the European ADR database EudraVigilance and the database of the vARIANCE study.

**Methods:**

The cohort of the vARIANCE study comprised 114 patients who suffered from ACEi‐ or ARB‐induced angioedema. In addition, 171 angioedema reports and 4650 reports on other ADRs of ACEi/ARB were identified in the ADR database EudraVigilance with the latter serving as a reference group. Odds ratios were calculated and a logistic regression analysis was performed using angioedema versus reference reports.

**Results:**

Increased age, smoking, allergies and a history of previous angioedema were identified as associated factors for ACEi‐/ARB‐induced angioedema. In most patients, the swelling affected the face, lips and tongue. In the vARIANCE study, about 70% of angioedema occurred after 1 year of treatment. For one in two patients in the vARIANCE study (84.2% with ACEi treatment) and one in three patients from the EudraVigilance reports (59.6% with ARB treatment), the angioedema resulted in hospitalization.

**Conclusions:**

We found small to moderate associations with certain individual patient‐related factors in this pharmaco‐epidemiological study. As a future perspective, combining non‐genetic association factors with corresponding genetic data might provide an option to compose stronger and individual risk scores.

## 1 INTRODUCTION

Angiotensin‐converting enzyme inhibitor (ACEi)‐ or angiotensin receptor blocker (ARB)‐induced angioedema is a rare adverse drug reaction (ADR) resulting in a potentially life‐threatening subcutaneous swelling of, for example, the face, larynx or pharynx.^1^, ^2^ In Germany, ACEi and ARBs are the most commonly administered drug classes, with 6017 million defined daily doses (DDD) and 4599 million DDD, respectively.^3^ These two drug classes accounted for a quarter of all prescribed daily doses in 2021. Thus, despite its rare occurrence, the widespread use of these drugs leads to a relevant number of patients developing ACEi‐ or ARB‐induced angioedema. Approximately one‐third of all angioedema cases admitted to emergency departments are caused by ACEi.^4^ Although most of these angioedema episodes are mild, angioedema often occurs in the head and neck area^5^ and therefore poses a potential risk of suffocation if there is progression to airway obstruction.^6^

The exact mechanisms underlying the development of ACEi‐ and ARB‐induced angioedema are not fully understood; however, the accumulation of bradykinin is thought to be a central factor.^7^, ^8^, ^9^ In addition, both a genetic predisposition and environmental factors are thought to determine a patient's individual susceptibility. Factors found to be associated with ACEi‐induced angioedema are African American descent^10^, ^11^ and female sex due to increased levels of oestrogen,^1^, ^12^, ^13^ although in the basic cohort of one analysis there was no association with female sex.^14^ With higher age and decreasing levels of dipeptidyl peptidase IV (DPP‐IV),^15^ the likelihood of developing angioedema increases.^11^, ^14^, ^16^ Other factors include a history of drug rash, seasonal allergies^2^, ^11^ and smoking.^2^, ^17^ Certain drugs have been associated with an increased risk of developing ACEi‐induced angioedema such as nonsteroidal anti‐inflammatory drugs (NSAID)^4^ and immunosuppressant drugs.^18^ A history of previous angioedema^19^ has been reported to increase the risk of ACEi‐induced angioedema. In contrast, diabetes was described to be conversely associated with ACEi‐induced angioedema.^1^, ^11^, ^20^

ARB‐induced angioedema is reported to occur slightly less than half as often as ACEi‐induced angioedema and far less data is available.^21^, ^22^ Information about phenotypes and occurrence of ARB‐induced angioedema is rare.

Risk factors such as a history of allergy and urticaria as an ADR were reported more frequently for ARB‐ than ACEi‐induced angioedema.^23^ In summary, the literature is inconsistent with regard to the associated factors of ACEi‐ and ARB‐induced angioedema.

The aim of our analysis was to further investigate non‐genetic association factors of ACEi‐/ARB‐induced angioedema using two different data sources: the European ADR database EudraVigilance and the database of the vARIANCE study. Since the nature of data differs between these two data sources, we assumed that an analysis of both databases would provide a more complete picture than an analysis of only one of these.

## 2 MATERIAL AND METHODS

### 2.1 EudraVigilance

The ADR database EudraVigilance of the European Medicines Agency (EMA)^24^ includes all spontaneously reported ADRs by health care professionals (HCP) such as physicians and pharmacists or non‐health care professionals (non‐HCP) such as patients and their relatives from the member states of the European Economic Area. In EudraVigilance, ADRs are coded in accordance with MedDRA terminology^25^ and drugs in accordance with the EudraVigilance medicinal product dictionary.^26^ MedDRA terminology is a standardised medical terminology with five different hierarchical levels to code, among others, diagnoses, laboratory results and symptoms. The structure of the five different hierarchical levels enables specific and aggregated analyses of the reported ADRs. Among others, the preferred term (PT) level describes the symptoms, laboratory results or diagnoses and the system organ class (SOC) level the groupings of these by aetiology, manifestation site or purpose. Furthermore, standardised MedDRA queries (SMQ)^27^ are pre‐determined sets of MedDRA terms (e.g. symptoms) grouped together to identify ADR reports describing specific diagnoses.

#### 2.1.1 Identification of angioedema reports

In this study, we identified all spontaneously reported cases of angioedema from Germany received between 01/01/2018 and 31/01/2023, in which an ACEi, ARB or their combination products were reported as suspected/interacting using the SMQ ‘angioedema (narrow)’ (*n* = 512)^27^ (Supporting Information S1: Appendix 1). We restricted the dataset to reports originating from HCPs (*n* = 270) in order to match the criteria of the vARIANCE study reports. Additionally, reports in which urticaria was coded were excluded (*n* = 46). The remaining 224 reports were assessed individually with regard to the causal relationship between the intake of the ACEi/ARB coded as suspected/interacting and the occurrence of the angioedema in accordance with the WHO criteria.^28^ Only reports with an at least possible causal relationship were considered for further analysis (*n* = 171). The completeness of the structured information provided in these ADR reports was measured by the vigiGrade completeness score.^29^ This score was originally established for the use in VigiBase, the ADR database of the WHO, and was thus slightly adapted to meet the structure of ADR reports from EudraVigilance. The analysed dataset (*n* = 171) achieved a median vigiGrade completeness score of 0.5 [0.4–0.9].

#### 2.1.2 Identification of reference reports

For the reference group, we identified all spontaneous reports from HCPs from Germany received between 01/01/2018 and 31/01/2023, in which an ACEi or ARB was coded as suspected/interacting. We excluded reports describing an angioedema according to the SMQ ‘angioedema (narrow)’ (*n* = 4800). Additionally, we excluded all reports referring to patients younger than 16 years (to align with the age of the patients in our angioedema reports), reports related to drug exposure during pregnancy (e.g. received from embryotox^30^), as well as reports with an unknown age, if the reported height and weight of the patients suggested that it was a child (*n* = 4662). After exclusion of duplicates 4650 reports remained. Due to the high number, these reports were not assessed individually.

### 2.2 vARIANCE study

The vARIANCE Study (Angioedema Risk Under Angiotensin‐Converting Enzyme Inhibitors) aims to identify factors associated with ACEi‐/ARB‐induced angioedema.^31^ The patients were recruited in Germany and Austria in several hospitals and primary care centres.^32^ The data were collected using a specific questionnaire. A more detailed description of the study can be found on the study website (www.variance‐studie.info). All participants provided written informed consent prior to inclusion, and the study was approved by the respective ethics committees.

#### 2.2.1 Identification of angioedema cases in the vARIANCE study

**FIGURE 1:**
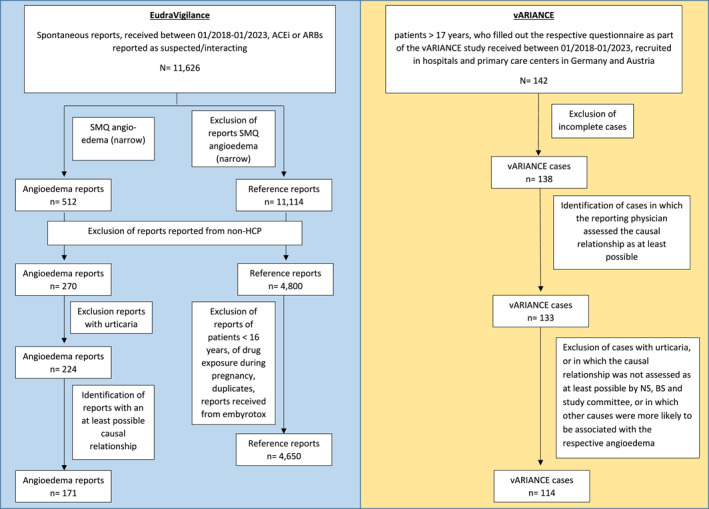
Identification of ADR reports in EudraVigilance and cases from the vARIANCE study.

All cases of adults aged 18–90 years received between 01/01/2018 and 31/01/2023 were identified in the database of the vARIANCE study (*n* = 142). Among these, cases with a complete questionnaire and an at least possible causal relationship between the intake of an ACEi or ARB, and the occurrence of angioedema according to WHO criteria,^28^ assessed by the reporting physician and the study committee, were included. Cases with self‐reported urticaria or other causes more likely to be associated with the angioedema were excluded. The final dataset consisted of *n* = 114 cases (Figure 1).

In 80 of these 114 cases (70.2%), the presence of hereditary angioedema (HAE) could be ruled out with high probability by screening either available sequencing data on five HAE‐associated genes (*SERPING1*, *F12*, *PLG*, *ANGPT1*, *KNG1*; *n* = 64, 80.0%)^32^ or relevant laboratory parameters (*n* = 16, 20.0%). However, cases of HAE with normal C1 esterase inhibitor concentration and activity may have been missed due to their normal C1 esterase inhibitor values and since not all to date known genes (e.g. *MYOF*, *HS3ST6*, *CPN1*) associated with this rare condition were investigated.

### 2.3 Drug prescription data

Prescription data according to 300 SGB V were provided by the Central Research Institute for Ambulatory Health Care in Germany^33^ for patients ≥16 years with at least one prescription of an ACEi, ARB or their combination products between 01/01/2018 and 30/06/2022. The number of drug prescriptions represents the total number of out‐patient prescriptions for patients with statutory health insurance (almost 90% of the German population^34^) dispensed in German pharmacies. Notably, in‐patient prescriptions are not covered.

### 2.4 Analysis criteria

#### 2.4.1 Analysis criteria for the descriptive analyses

The angioedema and reference reports from EudraVigilance and the cases from the vARIANCE study were analysed separately. The descriptive analyses included the patients' demographics, life‐style factors (e.g. smoking), previous patients' histories, previous angioedema, seriousness of reports, most frequently reported suspected/interacting ACEi/ARB, concomitant drugs and reported ADRs.

The seriousness in accordance with the legal definition^35^ could only be evaluated for the EudraVigilance reports. An ADR report is thus classified as serious if the ADR was life‐threatening, led to death, permanent disabilities, congenital anomalies or hospitalization or a prolongation thereof. This definition does not correspond to the clinical definition of the severity of an ADR. Please note that more than one seriousness criterion can be reported per ADR report.

In EudraVigilance, patients' histories and ADRs were analysed on the PT level of MedDRA terminology.^27^

### 2.5 Statistical analyses

#### 2.5.1 Descriptive analyses

R version 4.3.2 was used for statistical analyses.^36^ Means with standard deviations (SD) and medians with interquartile ranges (IQR) were calculated for patients' age and patients' body mass index (BMI). In EudraVigilance, means with SD and medians with IQR were also calculated for the number of days of the duration of ACEi/ARB treatment and persistent angioedema. All other results are presented as absolute numbers with their frequency distributions in the respective datasets.

#### 2.5.2 Calculation of reporting rates

The number of angioedema reports was divided by the number of ACEi/ARB prescriptions in order to calculate so‐called reporting rates. These were calculated for ACEi/ARBs, per year, for the individual drugs and for females and males. In order to improve robustness, the calculation of the reporting rates for the individual drugs was restricted to the drugs with more than three ADR reports. The reporting rates are presented as the number of angioedema reports per 10,000,000 prescription. Notably, the reporting rates only in the presence of the number of angioedema reports provided by HCP.

#### 2.5.3 Comparative analyses of angioedema and references reports from EudraVigilance

##### Odds ratio calculation

Odds ratios (OR) with their 95% confidence intervals (CI) were calculated using a two‐by‐two table to identify patients' histories, ADRs and concomitant drugs that were reported more frequently in angioedema than in reference reports.

##### Logistic regression analysis

Logistic regression analysis was carried out using angioedema versus reference reports as outcome variables and age, sex, seriousness, life‐style factors, selected patients' histories and selected concomitant drugs as covariates. Results obtained from logistic regression analyses are reported in terms of adjusted (adj.) OR with 95% CI. Selected patients' histories were chosen based on the diseases queried in the questionnaire of the vARIANCE study. The concomitant drugs were selected based on the evidence reported in the literature.^23^

##### Matching and conditional logistic regression analysis

A propensity score matching of each angioedema report to 10 reference reports based on patients' age and sex as well as the intake of an ACEi or ARB was performed using the MatchIt package in R.^37^ To this purpose, a nearest neighbour matching with a 1:10 ratio was used. The matching was found to be well balanced (Supporting Information S2: Appendix 2). Afterwards, conditional logistic regression analysis was carried out. Results are shown as adj. OR with 95% CI.

##### Interpretation of (adjusted) OR and 95% CI

If the lower CI was >1.0, we assumed that the respective information was more frequently reported in angioedema reports. If the upper CI was <1.0, we assumed that the respective information was more frequently reported in reference reports. No adjustment for multiple testing was performed.

## 3 RESULTS

### 3.1 Descriptive analyses of the patient populations

Patients who developed angioedema were on average 67 years old in EudraVigilance reports and 64 years old in the vARIANCE study and a slightly higher proportion were males (EudraVigilance: 52.0%; vARIANCE: 55.3%) (Table 1). Likewise, the BMI of the patients was on average higher than 25 (‘overweight’) in both datasets (EudraVigilance: 70.8%, vARIANCE: 77.5%). Most of the patients in the vARIANCE study with information about their self‐reported ancestry were Europeans (93.6%).

**TABLE 1:** Descriptive analyses of the patient populations.

	Angioedema cases vARIANCE study (*n* = 114)	Angioedema reports EudraVigilance (*n* = 171)
Demographical parameters of the patients		
Age		
Information reported	96.5% (*n* = 110)^a^	76.6% (*n* = 131)
Mean (+/−SD)	64.2 (+/−11.3)	67.5 (+/−14.2)
Median [IQR]	64.0 [55.0–72.0]	70.0 [58.0–78.5]
Sex		
Female	44.7% (*n* = 51)	47.4% (*n* = 81)
Male	55.3% (*n* = 63)	52.0% (*n* = 89)
Unknown	0.0% (*n* = 0)	0.6% (*n* = 1)
BMI		
Information reported	97.4% (*n* = 111)	38.0% (*n* = 65)
Mean (+/−SD)	29.5 (+/−6.2)	28.4 (+/−5.4)
Median [IQR]	28.4 [25.7–32.0]	26.6 [24.7–30.9]
Ancestry		
Information reported	96.5% (*n* = 110)	NA
European	93.6% (103/110)	NA
Asian	2.7% (3/110)	NA
African	1.8% (2/110)	NA
Others	1.8% (2/110)	NA
Life‐style factors of the patients^b^		
Alcohol consumption	52.6% (*n* = 60)	4.1% (*n* = 7)
Current smoker	16.7% (*n* = 19)	4.1% (*n* = 7)
Former smoker	48.2% (*n* = 55)	0.6% (*n* = 1)
Allergies and intolerances of the patients^c^		
Allergies reported	34.2% (*n* = 39)	9.9% (*n* = 17)
Intolerances reported	NA	2.3% (*n* = 4)
Summarized	34.2% (*n* = 39)	11.7% (*n* = 20)
The three most frequently reported allergies/intolerances	48.7% pollen/dust mites (19/39) 33.3% drugs (13/39) 20.5% food (8/39)	50.0% drugs (10/20) 35.0% pollen/dust mites (7/20) 15.0% food (3/20)
Previous angioedema in the histories of the patients		
Previous angioedema reported	57.9% (*n* = 66)	8.8% (*n* = 15)
Number of previous angioedema		
Once	16.7% (11/66)	NA
2–5 times	45.5% (30/66)	NA
6–10 times	15.2% (10/66)	NA
>10 times	18.2% (12/66)	NA
Previous angioedema related to drugs	69.7% (46/66)	NA
Previous angioedema related to ACEi/ARBs	63.6% (42/66)	73.3% (11/15)
Previous angioedema related to other causes	19.7% (13/66)	NA
Most frequently reported other causes		
Food	61.5% (8/13)	NA
Operation	23.1% (3/13)	NA
Stress	23.1% (3/13)	NA
Infection	15.4% (2/13)	NA
Histories of the patients^d^		
History reported	80.7% (*n* = 92)	64.9% (*n* = 111)
The three most frequently reported patient histories	87.0% hypertension (80/92) 25.0% diabetes mellitus type 2 (23/92) 19.6% hypothyroidism (18/92)	51.4% hypertension (57/111) 11.7% diabetes mellitus type 2 (13/111) 10.8% coronary artery disease (12/111)
Seriousness of angioedema reports/cases^e^		
Serious	NA	67.3% (*n* = 115)
Death	NA	2.9% (*n* = 5)
Life‐threatening	25.4% (*n* = 29)	10.5% (*n* = 18)
Hospitalisation	51.8% (*n* = 59)	31.0% (*n* = 53)
Disabling	NA	0.6% (*n* = 1)
Ambulance	45.6% (*n* = 52)	NA

*Note*: Descriptive analyses of the patients populations included in angioedema cases from the vARIANCE study and angioedema reports from EudraVigilance.

Abbreviations: ACEi, angiotensin‐converting enzyme inhibitor; ARB, angiotensin receptor blocker; BMI, body mass index; IQR, interquartile range; NA, information was not available in the respective dataset; SD, standard deviation.

^a^ In 4 patients of the vARIANCE study, the year of birth was reported, but the date of angioedema occurrence was missing. For this reason, no specific age value could be calculated for these patients. However, these patients were 18–90 years old according to the inclusion criteria of the vARIANCE study.

^b^ Note that information regarding consumption of alcohol and smoking as well as their regularity was specifically queried in the questionnaire of the vARIANCE study, whereas this information is not queried and only reported on a voluntary basis by HCP in the spontaneous reports. Thus, information concerning consumption of alcohol and smoking was less often provided in the spontaneous reports, which provides an explanation for the noticeable difference regarding their percentage in the vARIANCE study and the spontaneous reports.

^c^ More than one allergy or intolerance can be reported per patient.

^d^ In the vARIANCE study, some specific diseases were specifically queried in the questionnaire. In EudraVigilance, no specific diseases are queried. The reported histories in EudraVigilance were analysed on the PT level of MedDRA terminology.

^e^ In the vARIANCE study, the reporter could specify whether the respective angioedema was life‐threatening and led to hospitalization or visit of an ambulatory care centre/emergency treatment. In EudraVigilance, the classification of seriousness of reports follows the legal definition of seriousness. In EudraVigilance, a report is classified as serious if the reported ADR was life‐threatening, led to death, hospitalization or prolongation thereof, congenital anomalies or permanent disability.

In the vARIANCE study, about half of the patients reported consumption of alcohol (52.6%) and that they previously smoked (48.2%). Only 16.7% stated that they were current smokers. In contrast, only 4.1% of the patients in the EudraVigilance reports reported consumption of alcohol and current smoking, respectively, and one person was known to have smoked in the past (0.6%). In both datasets, former and current smoking as well as consumption of alcohol were more often reported for males than for females (Supporting Information S3: Appendix 3).

About one third of the patients from the vARIANCE study reported an allergy (34.2%) (Table 1). Most of them had allergies to pollen and dust mites (48.7%). Allergies against drugs were present in one third of these patients (33.3%). Allergies and intolerances were reported in 11.7% of the patients in EudraVigilance reports. Among these, allergies against drugs (50.0%) and pollen and dust mites (35.0%) were also reported most frequently.

A previous angioedema was known for 57.9% and 8.8% of the patients from the vARIANCE and the EudraVigilance datasets, respectively. Most of them were attributed to ACEi/ARBs (vARIANCE: 63.6%; EudraVigilance: 73.3%).

Chronic diseases were reported by 80.7% of the patients from the vARIANCE study, and by 64.9% of the patients from EudraVigilance. In both datasets, hypertension and type 2 diabetes mellitus ranked first and second among the chronic diseases. In EudraVigilance reports, coronary artery diseases ranked second among the chronic diseases most frequently reported in males (Supporting Information S3: Appendix 3).

About two‐thirds of the EudraVigilance reports were classified as serious (67.3%), in 31.0% a hospitalization or a prolongation thereof was mentioned, and in 5 reports (2.9%) a fatal outcome was reported. Slightly more than half of the patients from the vARIANCE study were hospitalized due to the angioedema (51.8%). In both datasets, angioedema seemed to be more serious in males than in females (Supporting Information S3: Appendix 3). In EudraVigilance reports, 55.9% of ARB‐ and 84.1% of ACEi‐induced angioedema were classified as serious (Supporting Information S4: Appendix 4).

### 3.2 Descriptive analyses of drug therapy

ACEi were reported as suspected in 84.2% of cases of the vARIANCE study and in 40.4% of the EudraVigilance reports (Table 2). In contrast, ARBs were mentioned as suspected in 59.6% of EudraVigilance reports compared to 11.4% of cases from the vARIANCE study. Ramipril (61.9%) was clearly most often stated as suspected ACEi/ARB in the vARIANCE study, followed by lisinopril (13.3%) and candesartan (9.7%). In the EudraVigilance reports, sacubitril/valsartan (29.8%) was the ACEi/ARB most frequently reported as suspected followed by ramipril (27.5%) and candesartan (17.5%). Sacubitril/valsartan was also the suspected drug most frequently reported for males in EudraVigilance reports, but ranked only third in reports for females (Supporting Information S2: Appendix 2).

**TABLE 2:** Descriptive analyses of ACEi/ARB therapy.

	Angioedema cases vARIANCE study (*n* = 114)	Angioedema reports EudraVigilance (*n* = 171)
Number of cases/reports related to ACEi or ARBs		
ACEi	84.2% (*n* = 96)	40.4% (*n* = 69)
ARB	11.4% (*n* = 13)	59.6% (*n* = 102)
ACEi and ARB	4.4% (*n* = 5)	0.0% (*n* = 0)
The five most frequently suspected ACEi/ARBs or their combination products^a^		
Information reported	99.1% (*n* = 113)	100.0% (*n* = 171)
1.	61.9% ramipril (70/113)	29.8% sacubitril/valsartan (51/171)
2.	13.3% lisinopril (15/113)	27.5% ramipril (47/171)
3.	9.7% candesartan (11/113)	17.5% candesartan (30/171)
4.	8.8% enalapril (10/113)	4.7% valsartan (8/171)
5.	2.7% valsartan (3/113)	3.5% enalapril (6/171)
Indication of ACEi/ARB therapy^b^		
Information reported	100% (*n* = 114)	73.7% (*n* = 126)
The three most frequently reported indications^a^		
1.	96.5% hypertension (110/114)	64.3% hypertension (81/126)
2.	3.5% cardiac insufficiency (4/114)	31.7% heart failure (40/126)
3.	3.5% cardiovascular prophylaxis (4/114)	2.4% coronary insufficiency (3/126)
Dose of reported suspected ACEi/ARB therapy		
Information reported	99.1% (*n* = 113)	66.6% (*n* = 114)
Standard	92.9% (105/113)	93.0% (106/114)
Increased	4.4% (5/113)	1.8% (2/114)
Decreased	2.7% (3/113)	5.3% (6/114)
Treatment duration of ACEi/ARB therapy until angioedema occurrence		
Information reported	95.6% (*n* = 109)	59.1% (*n* = 101)
1–3 days	3.7% (4/109)	27.7% (28/101)
4–14 days	4.6% (5/109)	12.9% (13/101)
>14 days—2 months	3.7% (4/109)	22.8% (23/101)
>2 months—1 year	14.7% (16/109)	15.8% (16/101)
>1 year	73.4% (80/109)	20.8% (21/101)
Mean number of days (+/−SD)	NA	478.5 (+/− 1086)
Median number of days [IQR]	NA	14.0 [3.0–300.0]
Duration of angioedema occurrence after exposure		
Information reported	95.6% (*n* = 109)	3.5% (*n* = 6)
<1 h	6.4% (7/109)	50.0% (3/6)
1–12 h	56.0% (61/109)	50.0% (3/6)
>12 h	37.6% (41/109)	0.0% (0/6)
Action taken with ACEi/ARB		
Information reported	99.1% (*n* = 113)	79.5% (*n* = 136)
Withdrawal	70.8% (80/113)	91.9% (125/136)
Dose reduced	0.0% (0/113)	0.7% (1/136)
Drug not withdrawn	29.2% (33/113)	7.4% (10/136)
Re‐exposure with the respective ACEi/ARB		
Information reported	88.6% (*n* = 101)	NA
Re‐exposure	31.7% (32/101)	NA
Angioedema after re‐exposure		
Yes	65.6% (21/32)	NA
No	25.0% (8/32)	NA
Unknown	9.4% (3/32)	NA

*Note*: Descriptive analyses of ACEi/ARB therapy in cases of the vARIANCE study and reports from EudraVigilance.

Abbreviations: ACEi, angiotensin converting enzyme inhibitors; ARB, angiotensin receptor blocker; NA, information was not available in the respective dataset.

^a^ Table 2 only represents the five most frequently reported ACEi and ARBs and the three most frequently reported drug indications. Note, that this is not a complete representation of all reported ACEi, ARBs and drug indications.

^b^ Note, that the terminologies used for the coding of drug indications differed between the vARIANCE study and EudraVigilance (e.g. cardiac insufficiency and heart failure). The reasoning is that in EudraVigilance (but not in the vARIANCE study) the coding of drug indications follows MedDRA terminology.

In both datasets, hypertension was the indication, and the standard dose was the dose most frequently reported (Table 2). Heart failure was reported as an indication in roughly one‐third of EudraVigilance reports compared to none of the cases from the vARIANCE study. Additionally, heart failure was reported as an indication in about half of the EudraVigilance reports with respective information referring to males and to ARBs (Supporting Informations S3 and S4: Appendices [Link], [Link]).

In the vARIANCE study, the duration of ACEi/ARB therapy until the occurrence of angioedema was longer than 1 year in 73.4% of the patients with respective information and only 12.0% of these patients developed angioedema in the first 2 months of drug therapy (Table 2). In contrast, in the EudraVigilance reports with respective information, angioedema occurred most frequently in the first 3 days of drug therapy (27.7%) and in nearly two‐thirds of all cases within the first 2 months. In only 20.8%, it occurred after one year (mean: 478.5 (+/−1086) days; median: 14 [3–300] days). The respective ACEi/ARB therapy was discontinued after the occurrence of angioedema in more than 70% of the patients with respective information in both datasets. However, in 29.2% of these patients in the vARIANCE study, the respective ACEi/ARB therapy was continued. In 31.7% of the patients from the vARIANCE study with respective information, re‐challenge was carried out and 65.6% of them developed angioedema again.

### 3.3 Descriptive analyses of angioedema

Even if the proportions differed clearly, lips, face and tongue were the locations most frequently affected by the angioedema in both datasets (Table 3). The angioedema lasted for 0–3 days in 83.2% of the patients from the vARIANCE study and 59.5% of the EudraVigilance reports (median: 3 [1–5] days) with respective information. Infections and substitution by a different brand with the same active ingredient were the most frequently reported associated factors in the vARIANCE study and EudraVigilance reports. Corrective treatment of angioedema was initiated in 83.3% of the patients in the vARIANCE study and in 20.8% of the patients in EudraVigilance reports. In both datasets, antihistamines and corticosteroids were most frequently used for the treatment of angioedema. In 55.8% and 25.5% of these patients from the vARIANCE study and from EudraVigilance reports, the treatment was considered effective.

**TABLE 3:** Descriptive analyses of angioedema features in the vARIANCE study and in EudraVigilance reports.

	Angioedema cases vARIANCE study (*n* = 114)	Angioedema reports EudraVigilance (*n* = 171)
The five most frequently reported locations of angioedema^a^ ^,^ ^b^		
Information reported	100.0% (*n* = 114)	100.0% (*n* = 171)
1.	71.1% lips (81/114)	28.1% tongue (48/171)
2.	54.4% face (62/114)	25.7% face (44/171)
3.	45.6% tongue (52/114)	25.1% lip (43/171)
4.	27.2% throat (31/114)	7.6% eyes (13/171)
5.	22.8% oral mucosa (26/114)	7.0% pharynx (12/171)
Duration of angioedema		
Information reported	99.1% (*n* = 113)	24.6% (*n* = 42)
<1 day	36.3% (41/113)	14.3% (6/42)
1–3 days	46.9% (53/113)	45.2% (19/42)
>3 days	16.8% (19/113)	40.5% (17/42)
Mean number of days (+/−SD)	NA	8.1 (+/−19.2)
Median number of days [IQR]	NA	3.0 [1.3–5.0]
Analysis of associated factors		
Associated factors reported	14.9% (*n* = 19)	25.1% (*n* = 43)
Most frequently reported associated factors^a^	41.2% infection (7/19) 29.4% stress (5/19) 11.8% food (2/19) 11.8% operation (2/19)	23.3% product substitution issue (10/43) 9.3% infection (4/43) 7.0% everolimus (3/43) 7.0% vaccination (3/43)
Corrective treatment of angioedema		
Corrective treatment of angioedema received^a^	83.3% (*n* = 95)	29.8% (*n* = 51)
Antihistamines	60.0% (57/95)	84.3% (43/51)
Corticosteroids	78.9% (75/95)	78.4% (40/51)
Epinephrine	1.1% (1/95)	17.6% (9/51)
C1‐esterase inhibitors	0.0% (0/95)	7.8% (4/51)
Icatibant	0.0% (0/95)	7.8% (4/51)
Response to corrective treatment reported	55.8% (53/95)	25.5% (13/51)
Immediately	NA	15.4% (2/13)
<6 h	NA	30.8% (4/13)

^a^ More than one localisation of angioedema, associated factor and corrective treatment could be reported per reported case.

^b^ Only the five most frequently reported localisations of angioedema are shown.

### 3.4 Comparative analyses of angioedema and reference reports from EudraVigilance

#### 3.4.1 Odds ratio calculation by two‐by‐two table

Compared to the reference group of all other ADR reports referring to ACEi/ARBs, everolimus (OR 41.5 [6.9–250.0]) was one of the concomitant drugs (descriptive analysis of reference reports see Supporting Information S5: Appendix 5, OR results see Supporting Information S6: Figure 1 of Appendix 6) and seasonal allergies (OR 3.6 [1.5–8.6]) and coronary artery diseases (OR 1.9 [1.1–3.6]) were two of the patient's histories (Supporting Information S6: Figure 2 of Appendix 6) more frequently reported in angioedema reports. In addition, some typical accompanying symptoms of angioedema such as stridor were more often described in angioedema versus reference reports (Supporting Information S6: Figure 3 of Appendix 6).

#### 3.4.2 Logistic regression analysis

The results of the logistic regression analysis revealed that reports of angioedema were classified as serious more often than the reference reports (adj. OR 2.2 [1.5–3.2]). Also, the presence of allergies (adj. OR 2.9 [1.4–5.8]), autoimmune disorders (adj. OR 2.8 [1.1–7.1]) and the concomitant treatment with fibrinolytics (adj. OR 16.4 [1.1–266.1]) were reported more frequently compared to the reference. However, the latter finding was based on only one case in both groups (Table 4).

**TABLE 4:** Logistic regression analysis and conditional logistic regression analysis for angioedema and reference reports.

EudraVigilance	Angioedema reports (*n* = 131)^a^	Reference reports (*n* = 2946)^a^	Logistic regression analysis: Adjusted OR [+/−95% CI]	Matched control (1:10) (*n* = 1310)^b^	Conditional logistic regression analysis: Adjusted OR [+/−95% CI]
Demographical parameters of the patients					
Mean age (+/− SD)	67.5 (14.2)	69.1 (13.5)	1.0 [1.0–1.0]	67.7 (+/−14.1)	‐
Female sex	67 (51.1%)	1462 (49.6%)	1.2 [0.8–1.7]	660 (50.4%)	‐
Classification of seriousness of ADR reports^c^					
Serious	88 (67.2%)	1537 (52.2%)	2.2 [1.5–3.2]	661 (50.5%)	‐
Life‐style factors^d^					
Smoker^e^	4 (3.1%)	56 (1.9%)	0.7 [0.2–2.5]	21 (1.6%)	0.6 [0.2–2.1]
Alcohol consumption^f^	5 (3.8%)	30 (1.0%)	2.5 [0.9–7.5]	13 (1.0%)	2.9 [0.8–9.7]
Histories of the patients^d^					
Atopic conditions^g^	3 (2.3%)	37 (1.3%)	0.9 [0.2–3.4]	17 (1.3%)	0.9 [0.2–3.6]
Allergy^h^	13 (9.9%)	101 (3.4%)	2.9 [1.4–5.8]	48 (3.7%)	3.3 [1.6–7.1]
Asthma^i^	3 (2.3%)	46 (1.6%)	1.1 [0.3–3.8]	20 (1.5%)	1.2 [0.3–4.3]
COPD^j^	6 (4.6%)	55 (1.9%)	2.2 [0.9–5.5]	31 (2.4%)	1.9 [0.7–4.6]
Diabetes^k^	12 (9.2%)	378 (12.9%)	0.6 [0.3–1.0]	158 (12.1%)	0.8 [0.4–1.5]
Hypothyroidism^l^	9 (6.9%)	378 (12.9%)	0.4 [0.2–0.8]	153 (11.7%)	0.5 [0.3–1.1]
Hyperthyroidism^m^	1 (0.8%)	21 (0.7%)	1.0 [0.1–7.5]	7 (0.5%)	1.3 [0.2–11.2]
Autoimmune diseases^n^	6 (4.6%)	41 (1.4%)	2.8 [1.1–7.1]	41 (3.1%)	1.6 [0.6–4.0]
Comedication^o^					
mTORi	2 (1.5%)	9 (0.3%)	4.3 [0.9–20.7]	5 (0.4%)	3.6 [0.7–18.1]
Fibrinolytics	1 (0.8%)	1 (0.0%)	16.4 [1.0–266.1]	1 (0.1%)	10.6 [0.7–172.1]
DPPIV inhibitors	4 (3.1%)	103 (3.5%)	0.7 [0.2–1.9]	40 (3.1%)	0.8 [0.3–2.5]

*Note*: Logistic regression analysis and the conditional logistic regression analysis of angioedema reports and reference reports form the EudraVigilance database. The respective life‐style factors, histories of the patients and comedications were selected because they were either specifically queried in the questionnaire of the vARIANCE study or were discussed in the literature as risk factors.

Abbreviations: COPD, chronic obstructive pulmonary disease; DPPIV inhibitors, dipeptidylpeptidase four inhibitors; SD, standard deviation.

^a^ Exclusive reports with unknown age, unknown sex and unknown seriousness.

^b^ Matched data in a 1:10 ratio taken into account age, sex, and ACEi or ARB intake.

^c^ The classification of seriousness of reports follows the legal definition of seriousness. In EudraVigilance, a report is classified as serious if the reported ADR was life‐threatening, led to death, hospitalization or prolongation thereof, congenital anomalies or permanent disabilities.

^d^ Appropriate PT or HLT levels of MedDRA terminology were used in order to identify the respective patients' histories and life‐style factors.

^e^ Smokers were identified by determining the number of reports with the HLT ‘Tobacco use’ exclusive of the terms related to ‘Non‐tobacco user’ of MedDRA terminology in the history of the patients.

^f^ Consumption of alcohol was identified by determining the number of reports with the HLT ‘Alcohol product use’ exclusive of the terms related to ‘Abstains from alcohol’ of MedDRA terminology in the history of the patients.

^g^ Atopic disorders were identified by determining the number of reports with the HLT ‘Atopic disorder’ of MedDRA terminology in the history of the patients.

^h^ Allergy was identified by determining the number of reports with the HLTs ‘Allergic conditions NEC’ and ‘Allergies to foods, food additives, drugs and other chemicals’ of MedDRA terminology in the history of the patients.

^i^ Asthma was identified by determining the number of reports with the PT ‘Asthma’ of MedDRA terminology in the history of the patients and reported as indication of drug therapy.

^j^ COPD was identified by determining the number of reports with the PT ‘Chronic obstructive pulmonary disease’ of MedDRA terminology in the history of the patients and reported as an indication of drug therapy.

^k^ Diabetes was identified by determining the number of reports with the HLT ‘Diabetes mellitus (incl. subtypes)’ of MedDRA terminology in the history of the patients and reported as indication of drug therapy. In addition, all patients with respective use of antidiabetic drugs excluding those using DPPIV inhibitors were identified.

^l^ Hypothyroidism was identified by determining the number of reports with the HLT ‘Thyroid hypofunction disorders’ of MedDRA terminology in the history of the patients and reported as indication of drug therapy. In addition, all patients with respective use of drugs for the treatment of thyroid hypofunction disorders were identified.

^m^ Hyperthyroidism was identified by determining the number of reports with the HLT ‘Thyroid hyperfunction disorders’ of MedDRA terminology in the history of the patients and reported as indication of drug therapy. In addition, all patients with respective use of drugs for the treatment of thyroid hyperfunction disorders were identified.

^n^ Autoimmune disorders were identified by determining the number of reports with the PTs included in the SMQ ‘immune‐mediated/autoimmune disorders (narrow)’ of MedDRA terminology in the history of the patients.

^o^ All drugs co‐reported to ACEi/ARBs were considered as concomitant drugs regardless of whether these were reported as suspected/interacting or concomitant. The respective drug classes were analysed in accordance with the ATC code.

#### 3.4.3 Conditional logistic regression analysis

After matching one angioedema to 10 reference reports taking into account age, sex and ACEi or ARB intake, only the presence of allergies (adj. OR 3.3 [1.6–7.1]) remained more frequently reported in angioedema versus reference reports (Table 4).

### 3.5 Number of angioedema reports from HCPs in relation to their number of drug prescriptions (=reporting rates)

The annual number of angioedema reports associated with ACEi/ARBs reported by HCPs decreased between 2018 and 2021 (from 46 to 19 reports, −41.3%), whereas the total number of ACEi/ARB prescriptions increased slightly (from ∼58 to ∼61 million, +4.9%). Summarized for 2018–2021, 4 angioedema reports per 10 million ACEi prescriptions and 7 angioedema reports per 10 million ARB prescriptions were reported by HCPs (Table 5). Considering the ACEi/ARBs with more than 3 reports from HCPs, the highest reporting rates per 10 million prescriptions were observed for sacubitril/valsartan (542 reports), followed by lisinopril (21 reports) and valsartan (18 reports).

**TABLE 5:** Number of reports per 10 million ACEi/ARB prescriptions (=reporting rates).

Period of analysis^a^: 01/2018–06/2021	Number of reports per 10 million prescriptions	Females: Number of reports per 10 million prescriptions	Males: Number of reports per 10 million prescriptions
Reporting rates^b^ of drug classes			
ACEi (*n* = 64)	4.4	4.2	4.6
ARB (*n* = 99)	6.8	6.9	6.7
Reporting rates^b^ of the suspected ACEi/ARBs with more than 3 reports			
Sacubitril/valsartan (*n* = 49)	541.6	102.3	155.8
Ramipril (*n* = 44)	14.7	4.6	4.8
Candesartan (*n* = 29)	15.6	7.7	1.3
Valsartan (*n* = 8)	17.6	8.3	3.6
Enalapril (*n* = 5)	14.2	0.0	8.5
Lisinopril (*n* = 5)	21.1	7.5	5.4

*Note*: Number of reports per 10 million prescriptions (=reporting rate) for the drug classes of ACEi and ARBs as well as for the individual ACEi/ARBs with more than 3 reports. Identification of ADR reports in EudraVigilance and cases from the vARIANCE study.

^a^ Drug prescription data were only available until 31/06/2021. Thus, the number of reports per drug class and for the individual ACEi/ARBs received between 01/01/2018 and 31/06/2021 was determined.

^b^ Reporting rates were calculated by dividing the number of reports for ACEi/ARBs or individual drugs by the number of prescriptions. In order to be more robust, the calculation of the reporting rates for the individual drugs was restricted to drugs with more than three ADR reports. The reporting rate is presented as the number of reports per 10 million prescription.

## 4 DISCUSSION

Our study represents a retrospective and descriptive analysis of reports of ACEi‐/ARB‐induced angioedema from the vARIANCE study and the EudraVigilance database.

### 4.1 Association with demographic parameters

The average age of the patients was 64.2 (+/−11.3) (vARIANCE) and 67.5 (+/− 14.2) (EudraVigilance) years which are consistent with other studies reporting that age >65 is a risk factor (OR 1,36 (KI 1.07–1.73)), but may also reflect the average age of the patients typically taking ACEi and ARB.^14^, ^16^, ^38^

In previous studies, female sex was found to be positively associated with ACEi‐induced angioedema probably due to increased levels of oestrogen.^13^, ^39^ In contrast, our study findings across both datasets consisted of slightly more cases reported in males. Likewise, 51% of the patients were males in a study by Mahmoudpour et al. (2016).^14^ Considering the number of reports per 10 million drug prescriptions from HCP from EudraVigilance, sex differences could only be observed for some individual ACEi/ARBs but not for the classes of ACEi and ARB themselves. Angioedema in males were more often classified as serious than in females in our analysis and thus may be reported more often.^40^ Further studies are needed to clarify whether males more often develop serious angioedema than females.

### 4.2 Association with allergy

The proportion of patients with allergies in the vARIANCE study (34.2%) was roughly similar to the proportion expected in the German population (females: 34.7%; males 27.0%).^41^ In the conditional logistic regression analysis, allergy was clearly more often reported in angioedema compared with reference reports (adj. OR 3.3 [1.6–7.1]). Likewise, a history of drug rash and seasonal allergies was reported to be positively associated with ACEi‐induced angioedema by others.^11^

### 4.3 Association with underlying diseases

In both datasets, hypertension and type 2 diabetes mellitus were reported most frequently among chronic diseases. Hypertension is often treated with ACEi or ARBs, thus explaining the high number of reports on this comorbidity.^38^, ^42^ In Germany, a slightly higher prevalence of coronary artery diseases was observed for males than for females.^41^ Heart failure and coronary artery diseases may favour the occurrence of angioedema.^1^ Likewise, coronary artery diseases were reported most often for males in EudraVigilance reports suggesting a possibly higher risk to develop angioedema and probably explaining the slightly higher number of angioedema reports for males in general and of those classified as serious in our analysis.

Diabetes was reported to be negatively associated with ACEi‐ and ARB‐induced angioedema.^1^, ^11^ The tendency of a lower risk for patients with type 2 diabetes mellitus when comparing angioedema and reference reports may be assumed based on our results of logistic regression analysis (adj. OR 0.6 [0.3–1.0]).

More than two‐thirds of patients in both datasets were overweight. Overweight was associated with the re‐occurrence of angioedema and was expected to be a risk factor for more severe angioedema by others.^16^ However, differences in BMI could not be confirmed between angioedema and reference reports in our analysis.

### 4.4 Association with co‐medication

In a retrospective study, angioedema because of a combination of mTORi and ACEi was diagnosed in 6.6% (*n* = 9) versus 1.9% (*n* = 1) of patients with kidney transplants exposed to mTORi but not exposed to ACEi.^18^ Likewise, angioedema occurs 1.3%–5.9% (*n* = 1–15)^43^, ^44^, ^45^, ^46^, ^47^ more frequently when ACEi are concomitantly used with fibrinolytics compared to controls^48^ probably due to increased production of bradykinin.^43^ Adding to these observations, we found that everolimus and fibrinolytics were more frequently reported in angioedema compared to reference reports, albeit with wide confidence intervals due to small sample sizes (mTORi, *n* = 2 and fibrinolytics, *n* = 1, Table 4). The combination of ACEi with everolimus or alteplase is already listed as contraindication in the respective product information.^49^, ^50^

### 4.5 Association with life‐style factors

Smokers have an increased risk of developing angioedema due to significantly lower serum DPP‐IV activity.^20^ Likewise, alcohol‐related disorders may play a role in ACEi‐induced angioedema.^51^ In summary, however, we did not find a clear association of angioedema with current smoking status and alcohol consumption in our analysis. Such an association, however, could also be related to co‐morbidities such as coronary artery disease, heart failure, and hypertension. The number of cases with respective information in both datasets and the degree of information available did not allow investigation of possible interactions.

### 4.6 Previous angioedema

57.9% (vARIANCE) and 8.8% (EudraVigilance) of the patients had a known history of previous angioedema and more than two‐thirds of these were attributed to ACEi/ARBs. A 10‐fold increased risk of further angioedema has been reported with continuous ACEi treatment despite the occurrence of previous angioedema.^2^ Previous ACEi‐ or ARB‐induced angioedema are already listed as contraindications in the product information of ACEi and ARBs. The re‐occurrence of angioedema was also seen in our data, namely in two of three patients with angioedema after re‐exposure. Likewise, almost 90% of the patients in the vARIANCE study with a known history of angioedema developed more than one angioedema episode in the past (unknown if associated with ACEi/ARB therapy). However, it may be possible that the previous angioedema was not attributed to the ACEi/ARB intake since about three quarters of all angioedema in the vARIANCE study occurred after 1 year of drug therapy. Nevertheless, in almost one third of all patients (29.2%) from the vARIANCE study, the respective ACEi/ARB therapy was continued after angioedema occurrence, although the responses might partly result from a misunderstanding of the respective question in the questionnaire. As a translation into clinical practice, physicians should question the patients with regard to the occurrence of angioedema when filling a repeat‐prescription for ACEi or ARB.

### 4.7 Clinical phenotype and time to onset of angioedema

In both datasets, the face, lips and tongue were most frequently affected by angioedema, thereby confirming respective findings in the literature.^5^ In accordance with literature,^5^ we assume that there is no relevant difference between the phenotypes of ACEi‐versus ARB‐induced angioedema, since the clinical phenotypes did not differ between the vARIANCE study and the EudraVigilance reports.

Divergent results were observed concerning the time to onset of the angioedema. While in 70% of patients in the vARIANCE study, the angioedema occurred after 1 year of treatment, the angioedema occurred within the first year in about 80% of the EudraVigilance reports. According to the literature, ACEi‐induced angioedema most frequently develops within the first weeks of treatment but may occur at any time, even years after initiating ACEi therapy.^2^ Our findings in the vARIANCE study support the occurrence of angioedema later than a year from initiation, and this, as a clinical translation, is an aspect physicians should be aware of.

### 4.8 Severity and treatment of the angioedema cases

Overall, ACEi‐ and ARB‐induced angioedema were quiet serious, necessitating hospital admission in every second patient in the vARIANCE study and every third patient from the EudraVigilance reports. ACEi‐compared to ARB‐induced angioedema were reported as more serious and resulted in hospitalization or prolongation thereof more often.^23^, ^52^ This was also reflected in the EudraVigilance reports in our analysis.

In patients with respective information, antihistamines and corticosteroids were most frequently administered as a therapy, as also reported in the literature.^53^ Treatment with antihistamines and corticosteroids was considered to be effective in some cases, which may indicate possible effectiveness although stated otherwise.^2^, ^53^, ^54^ Another explanation could be the discontinuation of the ACEi/ARBs (especially for those lasting longer than 3 days) as well as the spontaneous remission, which could lead to an improvement of symptoms.

### 4.9 Proportion of ACEi‐versus ARB‐induced angioedema in both datasets

In Germany, ACEi is prescribed more frequently than ARB (Supporting Information S7: Appendix 7). With an incidence of 1.79 for ACEi and 0.62 for ARBs in 1000 people, ACEi‐induced angioedema is almost three times more common than ARB‐induced angioedema.^52^ A clearly higher proportion of ACEi‐ than ARB‐induced angioedema was also observed in the vARIANCE study but not in the EudraVigilance reports. The combination sacubitril/valsartan was the ARB most frequently reported in EudraVigilance reports. Other studies did not show a higher risk of angioedema associated with sacubtiril/valsartan compared with ACEi or ARBs.^55^, ^56^, ^57^, ^58^ Thus, reporting bias has to be considered.^40^

## 5 STRENGTHS AND LIMITATIONS OF THE STUDY

The strengths of our study are the complementary approach, the analysis of a systematically collected dataset (vARIANCE study) and a dataset of spontaneous reports (EudraVigilance), thereby providing a more complete picture compared to the analysis of one database alone.

One of the major limitations of the analyses of the spontaneous reports is the unknown amount of underreporting.^59^ In addition, differences between the datasets may also be related to reporting bias since physicians may tend to report serious ADRs or ADRs to newer drugs (e.g. sacubitril/valsartan) more often than non‐serious ADRs or those related to older drugs (e.g. ACEi). In case of seriousness, this may also apply to the vARIANCE cases, expecting more severe than mild cases of ACEi‐ and ARB‐induced angioedema to present to a dermatology/allergy or otorhinolaryngology department.

Obviously, the information contained in the EV reports is not as comprehensive (e.g., concerning life‐style factors) as the information received via a 10‐page questionnaire specifically adapted to collect relevant information about ACEi‐ and ARB‐induced angioedema in the vARIANCE study leading to a lower number of reports with information concerning specific categories.

Finally, we cannot completely rule out very rare cases of HAE among the vARIANCE cases.

## 6 CONCLUSION

In our study, we confirmed the positive and negative associations of several factors associated with ACEi‐ and ARB‐induced angioedema described earlier. However, most of these factors did not provide a strong enough positive association to pinpoint a distinct high‐risk group of patients or high‐risk constellations. Based on our results, physicians should be aware and consider that patients with previous angioedema to ACEi/ARBs carry a higher risk of angioedema (also listed as contraindication). In addition, we would not conclude that tolerance develops towards angioedema with continued therapy. Finally, non‐occurrence of angioedema in the first weeks of therapy is no re‐assurance for their continued absence, as they may occur after a longer time of treatment.

As a future perspective, the combination of individual pharmaco‐epidemiological data with corresponding genetic findings^60^ may help to identify patient groups or constellations carrying a distinct higher risk of ACEi‐ or ARB‐induced angioedema.

## AUTHOR CONTRIBUTIONS

**Diana Dubrall:** Conceptualization; formal analysis; investigation; writing—original draft; methodology; visualization. **Nora L. Branding:** Conceptualization; investigation; writing—review and editing; Project administration. **Carina M. Mathey:** Resources; writing—review and editing; project administration. **Anna M. Weber:** Writing—original draft; project administration. **Michael Steffens:** Writing—review and editing. **Maike Below:** Writing—review ‐ editing; resources. **Matthias Schmid:** Writing—review and editing. **Bettina Wedi:** Resources; writing—review and editing. **Dorothea Wieczorek:** Resources; writing—review and editing. **Philipp M. Amann:** Resources; writing—review and editing. **Harald Löffler:** Resources; writing—review and editing. **Lukas Koch:** Resources; writing—review and editing. **Clemens Schöffl:** Resources; writing—review and editing. **Heinrich Dickel:** Resources; writing—review and editing. **Nomun Ganjuur:** Resources; writing—review and editing. **Thorsten Hornung:** Resources; writing—review and editing. **Timo Buhl:** Resources; writing—review and editing. **Emel Aygören‐Pürsün:** Resources; writing—review and editing. **Gerda Wurpts:** Resources; writing—review and editing. **Jens Greve:** Resources; writing—review and editing. **Markus M. Nöthen:** Resources; writing—review and editing; funding acquisition; supervision. **Andreas J. Forstner:** Resources; writing—review and editing; supervision; funding acquisition. **Bernhardt Sachs:** Funding acquisition; resources; conceptualization; investigation; writing—original draft.

## CONFLICT OF INTEREST STATEMENT

MMN has received fees for membership in the advisory board from HMG Systems Engineering GmbH (Fürth, Germany) and in the Medical‐Scientific Editorial Office of the Deutsches Ärzteblatt, for review activities from the European Research Council (ERC), and for serving as a consultant for EVERIS Belgique SPRL in a project of the European Commission (REFORM/SC2020/029). MMN receives salary payments from Life & Brain GmbH and holds shares in Life & Brain GmbH.

## DISCLAIMER

The information and views set out in this manuscript are those of the authors and do not necessarily reflect the official opinion of the Federal Institute for Drugs and Medical Devices.

## Supporting information

Supporting Information S1

Supporting Information S2

Supporting Information S3

Supporting Information S4

Supporting Information S5

Supporting Information S6

Supporting Information S7

## Data Availability

Data sharing is not applicable to this article as no new data were created or analyzed in this study.
