# Supplementary material for: Non‐genetic factors associated with ACE‐inhibitor and angiotensin receptor blocker‐induced angioedema
Source: Clin Transl Allergy. 2025 May 7;15(5):e70058. doi: 10.1002/clt2.70058 (PMC12058302; doi:10.1002/clt2.70058)
Supplement: Supplementary file 1 — Supporting Information S1 [file CLT2-15-e70058-s006.docx]

**Appendix 1) Descriptive analyses of all angioedema reports identified by SMQ angioedema (narrow) and angioedema reports without urticaria.**

Appendix 1 Table 1) Descriptive analyses of all angioedema reports identified by SMQ angioedema (narrow) and angioedema reports without urticaria.

| **EudraVigilance reports** | **ACEi/ARBs: Angioedema reports with an at least possible causal relationship (n= 171)^1^** | **ACEi/ARBs: Angioedema reports identified by SMQ angioedema (narrow) (n= 512)** | **ACEi/ARBs: Angioedema reports identified by SMQ angioedema (narrow) exclusive reports with urticaria (n= 427)** |
| --- | --- | --- | --- |
| **Demographical parameters** | | |  |
| **Age**  **Reports with information**  **Mean (+/-sd)**  **Median [IQR]**  **Sex**  **Female**  **Male**  **Unknown**  **BMI**  **Reports with information**  **Mean (+/-sd)**  **Median [IQR]** | 76.6% (n= 131)  67.5 (+/-14.2)  70.0 [58.0-78.5]  47.4% (n= 81)  52.0% (n= 89)  0.6% (n= 1)  38.0% (n= 65)  28.4 (+/-5.4)  26.6 [24.7-30.9] | 64.8% (n= 332)  66.7 (+/-13.6)  69.0 [58.8-77.0]  52.9% (n= 271)  45.3% (n= 232)  1.8% (n= 9)  37.7% (n= 193)  28.6 (+/-5.6)  27.8 [24.7-31.3] | 65.6% (n= 280)  67.1 (+/- 13.3)  70.0 [59.0-77.0]  54.1% (n= 231)  44.3% (n= 189)  1.6% (n= 7)  37.2% (n= 159)  28.5 (+/- 5.7)  27.5 [24.7-31.2] |
| **The three most frequently reported histories (PT-level) of the patients^3^** | | |  |
| **History reported**  **1.**  **2.**  **3.** | 64.9% (n= 111)  51.4% hypertension (57/111)  11.7% type 2 diabetes mellitus (13/111)  10.8% coronary artery disease (12/111) | 61.1% (n= 313)  56.5% hypertension (177/313)  6.7% type 2 diabetes mellitus (21/313)  6.4% seasonal allergy (20/313) | 61.8% (n= 264)  56.4% hypertension (149/264)  8.0% type 2 diabetes mellitus (21/264)  6.1% coronary artery disease (16/264)  6.1% obesity (16/264)  6.1% seasonal allergy (16/264) |
| **Seriousness criteria of the ADR reports^4^** | | |  |
| **Serious**  **Death**  **Life-threatening**  **Hospitalisation**  **Disabling** | 67.3% (n= 115)  2.9% (n= 5)  10.5% (n= 18)  31.0% (n= 53)  6.6% (n= 1) | 48.6% (n= 249)  1.6% (n= 8)  35.1% (n= 26)  18.6% (n= 95)  0.8% (n= 4) | 54.1% (n= 231)  1.9% (n= 8)  6.1% (n= 26)  21.3% (n= 91)  0.7% (n= 3) |
| **Most five most frequently reported ACEi/ARBs reported as suspected/interacting^5^** | | |  |
| **Information reported**  **1.**  **2.**  **3.**  **4.**  **5.** | 100.0% (n= 171)  29.8% sacubitril/valsartan (51/171)  27.5% ramipril (47/171)  17.5% candesartan (30/171)  4.7% valsartan (8/171)  3.5% enalapril (6/171) | 100.0% (n= 512)  23.6% ramipril (121/512)  22.3% candesartan (114/512)  17.4% sacubitril/valsartan (89/512)  8.6% valsartan (44/512)  4.3% candesartan/HCT (22/512) | 100.0% (n= 427)  25.1% ramipril (107/427)  21.3% candesartan (91/427)  18.0% sacubitril/valsartan (77/427)  8.2% valsartan (35/427)  3.7% candesartan/HCT (16/427) |
| **The five most frequently reported ADRs (PT-level)^7^** | | |  |
| **Information reported**  **1.**  **2.**  **3.**  **4.**  **5.** | 100.0% (n= 171)  44.4% angioedema (76/171)  27.5% swollen tongue (47/171)  19.9% swelling face (34/171)  18.7% lip swelling (32/171)  12.3% dyspnea (21/171) | 100.0% (n= 512)  24.2% angioedema (124/512)  23.0% swelling face (118/512)  20.5% swollen tongue (105/512)  16.6% urticaria (85/512)  15.6% lip swelling (80/512) | 100.0% (n= 427)  28.1% angioedema (120/427)  25.5% swelling face (109/427)  23.4% swollen tongue (100/427)  17.8% lip swelling (76/427)  10.5% dyspnea (45/427) |

Appendix 1 Table 1 shows the descriptive analyses of all angioedema reports identified by SMQ „angioedema (narrow)“, angioedema reports excluding those with urticaria (n= 427) and angioedema report with an at least possible causal relationship according to WHO criteria.

Differences between the three datasets were observed with regard to the sex of the patients, seriousness of reports and the most frequently reported suspected ACEi/ARBs. Females were in proportion more often included in reports of all angioedema reports identified by SMQ “angioedema (narrow)” and angioedema reports without urticaria compared to angioedema reports with an at least possible causal relationship. Angioedema reports with an at least possible causal relationship were in proportion more often classified as serious as reports from the other two datasets. Sacubitril/valsartan was in proportion more frequently reported in angioedema reports with an at least possible causal relationship compared to the other two data sets. In the other two datasets, ramipril and candesartan ranked first followed by sacubitril/valsartan.
