# Supplementary material for: Non‐genetic factors associated with ACE‐inhibitor and angiotensin receptor blocker‐induced angioedema
Source: Clin Transl Allergy. 2025 May 7;15(5):e70058. doi: 10.1002/clt2.70058 (PMC12058302; doi:10.1002/clt2.70058)
Supplement: Supplementary file 2 — Supporting Information S2 [file CLT2-15-e70058-s004.docx]

**Appendix 2) Evaluation of propensity score matching of ACEi/ARB angioedema reports to reference reports**

Method: Each ACEi/ARB angioedema report with information concerning the age and sex of the patients (n= 131) was matched to 10 ACEi/ARB reference reports (n= 1,310) with regard to the criteria age, sex and ACEi or ARB intake.

The graphical evaluation of the matching by showing the distribution of propensity scores and the density plots of categories is presented below.

Appendix 2 Figure 1) Distribution of propensity scores


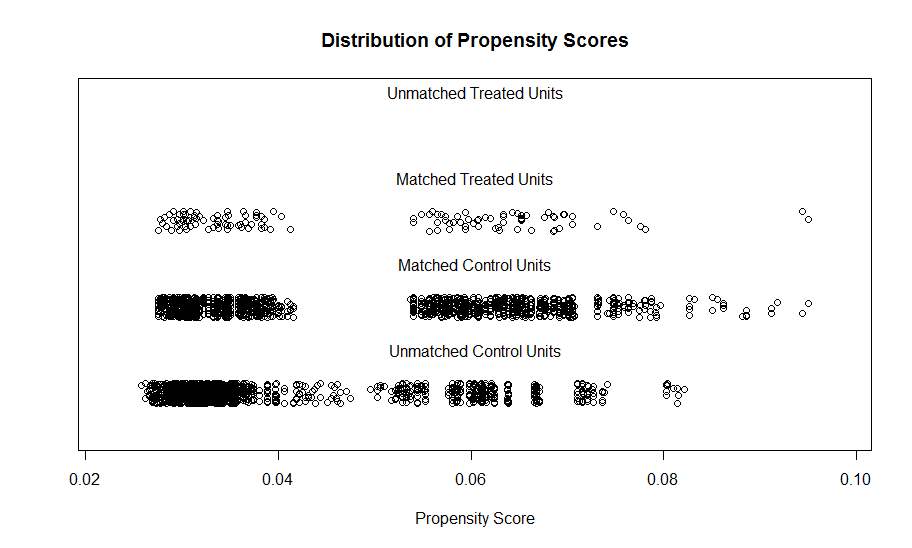


Appendix 2 Figure 1 shows the distribution of the propensity scores in the angioedema reports (=matched treated units), the matched reference reports of other ADRs to ACEi/ARB (= matched control units) and the unmatched reference reports of other ADRs to ACEi/ARB (= unmatched control units).

Appendix 2 Figure 2) Graphical evaluation of density plots of matching parameters


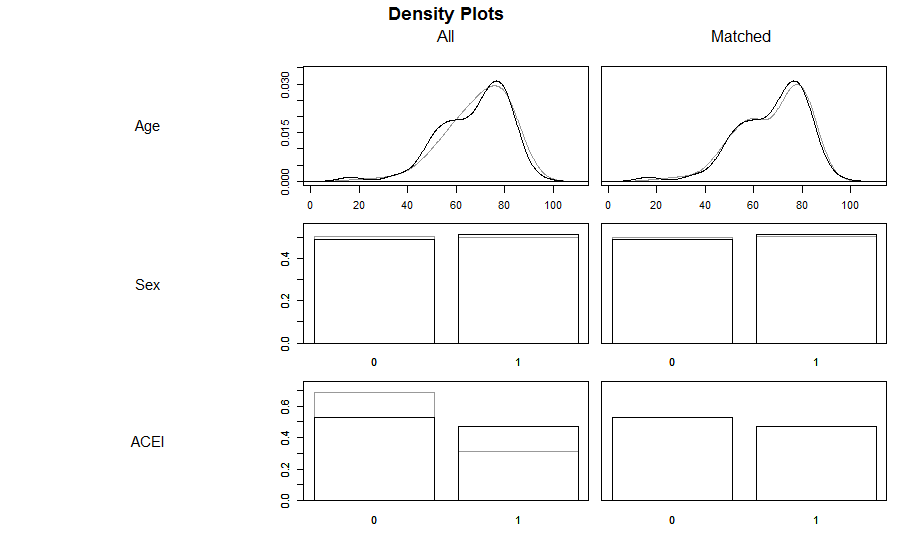


Appendix 2 Figure 2 shows the density plots of the matching parameters age, sex and ACEi or ARB intake before (= all) and after the matching procedure (= matched).
