# Supplementary material for: Non‐genetic factors associated with ACE‐inhibitor and angiotensin receptor blocker‐induced angioedema
Source: Clin Transl Allergy. 2025 May 7;15(5):e70058. doi: 10.1002/clt2.70058 (PMC12058302; doi:10.1002/clt2.70058)
Supplement: Supplementary file 3 — Supporting Information S3 [file CLT2-15-e70058-s003.docx]

**Appendix 3) Sex-stratified analyses of angioedema reports from EudraVigilance and angioedema cases from the vARIANCE study.**

Appendix 3 Table 1) Analyses of data of the patients included in angioedema reports and cases from EudraVigilance and the vARIANCE study.

|  | **Females: Angioedema cases vARIANCE study (n= 51)** | **Females: Angioedema reports EudraVigilance (n= 81)** | **Males: Angioedema cases vARIANCE study (n= 63)** | **Males: Angioedema reports EudraVigilance (n= 89)** |
| --- | --- | --- | --- | --- |
| **Demographical parameters of the patients** | |  |  |  |
| **Age**  **Information reported**  **Mean**  **Median**  **BMI**  **Information reported**  **Mean**  **Median**  **Ethnicity**  **Information reported**  **European/Caucasian**  **Asian**  **African**  **Others** | 94.1% (n= 48)  62.9 (+/-11.9)  62.5 [53.0-71.3]  96.1% (n= 49)  29.5 (+/-7.4)  28.3 [24.7-32.3]  96.1% (n 49)  95.9% (47/49)  0.0% (0/49)  0.0% (0/49)  4.1% (2/49) | 100.0% (n= 81)  66.3 (+/-15.0)  69.0 [58.0-78.5]  58.0% (n= 47)  27.3 (+/-4.9)  26.3 [24.6-29.7]  NA  NA  NA  NA  NA | 98.4% (n= 62)  65.2 (+/-10.7)  65.0 [58.3-72.0]  98.4% (n= 62)  29.4 (+/- 5.1)  28.5 [26.1-31.8]  96.8% (n= 61)  91.8% (56/61)  3.3% (2/61)  3.3% (2/61)  0.0% (0/61) | 100.0% (n= 89)  68.4 (+/-13)  70 [58-80]  65.2% (n= 58)  29.6 (+/-5.8)  27.6 [25.6-31.4]  NA  NA  NA  NA  NA |
| **Lifestyle factors of the patients** | |  |  |  |
| **Alcohol consumption**  **Current smoker**  **Former smoker** | 41.2% (n= 21)  13.7% (n= 7)  29.4% (n= 29) | 0.0% (n= 0)  0.0% (n= 0)  0.0% (n= 0) | 61.9% (n= 39)  19.0% (n= 12)  63.5% (n= 40) | 7.9% (n= 7)  7.9% (n= 7)  1.1% (n= 1) |
| **Allergies^1^** |  |  |  |  |
| **Allergies reported**  **Intolerances reported**  **The three most frequently reported allergies/intolerances** | 51.0% (n= 26)  46.2% pollen/dustmite  (12/26)  38.5% drugs  (10/26)  23.1% food  (6/26) | 9.9%% (n= 8)  3.7% (n= 3)  45.5% antibiotics (5/11)  18.2% nickel (2/11)  18.2% pollen/dustmite (2/11) | 20.6% (n= 13)  53.8% pollen/dustmite (7/13)  23.1% drugs (3/13)  15.4% food (2/13) | 9.0% (n= 8)  0.0% (n= 0)  62.5% pollen/dustmite (5/8)  25.0% antibiotics (2/8) |
| **Previous angioedema** | | | | |
| **Previous angioedema reported**  **Number of previous angioedema**  **Once**  **2-5 times**  **6-10 times**  **> 10 times**  **Previous angioedema related to drugs**  **Previous angioedema related to ACEi/ARBs**  **Previous angioedema related to other causes**  **Most frequently reported other causes**  **Food**  **Operation**  **Stress**  **Infection**  **Physical activity**  **Temperature (heat, cold)** | 60.8% (n= 31)  19.4% (6/31)  41.9% (13/31)  12.9% (4/31)  22.6% (7/31)  67.7% (21/31)  58.0% (18/31)  22.6% (7/31)  57.1% (4/7)  28.6% (2/7)  14.3% (1/7)  0.0% (0/7)  14.3% (1/7)  14.3% (1/7) | 7.4% (n= 6)  NA  NA  NA  NA  NA  100.0% (6/6)  NA  NA  NA  NA  NA  NA  NA | 56.5% (n= 35)  14.3% (5/35)  48.6% (17/35)  17.1% (6/35)  14.3% (5/35)  71.4% (25/35)  68.6% (24/35)  17.1% (6/35)  66.7% (4/6)  33.3% (2/6)  33.3% (2/6)  33.3% (2/6)  0.0% (0/6)  0.0% (0/6) | 10.1% (n= 9)  NA  NA  NA  NA  NA  55.6% (5/9)  NA  NA  NA  NA  NA  NA  NA |
| **Histories of the patients^2^** | | |  |  |
| **Histories reported**  **The three most frequently reported patient histories** | 88.2% (n= 45)  86.7% hypertension (39/45)  31.1% hypothyroidism (14/45)  15.6% type 2 diabetes mellitus (7/45)  15.6% asthma (7/45) | 66.7% (n= 54)  59.3% hypertension (n= 32/54)  9.3% type 2 diabetes mellitus (5/54)  9.3% drug hypersensitivity (5/54) | 74.6% (n= 47)  87.2% hypertension (41/47)  34.0% type 2 diabetes mellitus (16/47)  8.5% hypothyroidism (4/47) | 62.9% (n= 56)  42.9% hypertension (24/56)  16.1% coronary artery disease (9/56)  14.3% type 2 diabetes mellitus (8/56) |
| **Seriousness of angioedema reports/cases^3^** | | | | |
| **Serious**  **Death**  **Life-threatening**  **Hospitalisation**  **Disabling**  **Ambulance** | NA  NA  27.5% (n= 11)  51.0% (n= 26)  NA  42.0% (n= 21) | 60.5% (n= 49)  2.5% (n= 2)  12.3% (n= 10)  25.9% (n= 21)  0.0.% (n= 0)  NA | NA  NA  39.1% (n= 18)  52.4% (n= 33)  NA  49.2% (n= 31) | 74.2% (n= 66)  3.4% (n= 3)  9.0% (n= 8)  36.0% (n= 32)  1.1% (n= 1)  NA |

BMI= body mass index; SD= standard deviation; IQR= interquartile range; NA= information was not available in the respective data set; ACEi= angiotensin converting enzyme inhibitors; ARB= angiotensin receptor blocker

^1^ more than one allergy or intolerance can be reported per patient.

^2^ in the vARIANCE study some diseases were specifically queried in the questionnaire. In EudraVigilance, no specific diseases are queried. The reported histories in EudraVigilance were analysed on the PT-level of MedDRA terminology.

^3^ in the vARIANCE study the reporter could specify whether the respective angioedema was life-threatening, led to hospitalization or a visit of an ambulance. In EudraVigilance, the classification of seriousness of reports follows the legal definition of seriousness. In EudraVigilance a report is classified as serious if the reported ADR was life-threatening, led to death, hospitalisation or prolongation thereof, congenital anomalies or permanent disability.

Appendix 3 Table 1 shows the descriptive analyses of the patients populations included in angioedema cases referring to females and males from the vARIANCE study and angioedema reports from EudraVigilance.

Differences between females and males were observed with regard to life-style factors (smoking, consumption of alcohol), allergies (only vARIANCE study), histories of the patients (only EudraVigilance) and the seriousness of angioedema reports/cases. Smoking and consumption of alcohol was in proportion more frequently reported for males than for females in both data sets. Allergies were in proportion more frequently reported for females than males in the vARIANCE study. Coronary artery diseases were in proportion more frequently reported for males than for females in EudraVigilance reports. In both datasets, angioedema in males seemed to be more serious than in females.

Appendix 3 Table 2) Analyses of data concerning ACEi/ARB therapy in angioedema reports and cases from EudraVigilance and the vARIANCE study.

|  | **Females: Angioedema cases vARIANCE study (n= 51)** | **Females: Angioedema reports EudraVigilance (n= 81)** | **Males: Angioedema cases vARIANCE study (n= 63)** | **Males: Angioedema reports EudraVigilance (n= 89)** |
| --- | --- | --- | --- | --- |
| **The five most frequently suspected ACEi/ARBs** | | | | |
| **Information reported**  **1.**  **2.**  **3.**  **4.**  **5.** | 100.0% (n= 51)  62.7% ramipril (32/51)  13.7% enalapril (7/51)  5.9% candesartan (3/51)  3.9% lisinopril (2/51)  3.9% valsartan (2/51) | 100.0% (n= 81)  32.1% candesartan (26/81)  27.2% ramipril (22/81)  13.6 % sacubitril/valsartan (11/81)  7.4% valsartan (6/81)  3.7% lisinopril (3/81) | 98.4% (n= 62)  56.5% ramipril (35/62)  19.4% lisinopril (12/62)  9.7% candesartan (6/62)  4.8% enalapril (3/62)  3.2% lisinopril/HCT (2/62)  3.2% perindopril (2/62) | 100% (n= 89)  44.9% sacubitril/valsartan (40/89)  28.1% ramipril (25/89)  5.6% enalapril (5/89)  3.4% candesartan (3/89)  2.2% candesartan/HCT (2/89)  2.2% lisinoprol (2/89)  2.2% ramipril/HCT (2/89)  2.2% valsartan (2/89)  2.2% valsartan/HCT (2/89) |
| **The three most frequently reported indications of ACEi/ARB therapy** | | | | |
| **Information reported**  **1.**  **2.**  **3.** | 100% (n= 51)  96.1% hypertension (49/51)  3.9% cardiac insufficiency (2/51)  2.0% cardiovascular prophylaxis (1/51) | 72.8% (n= 59)  79.7% hypertension (47/59)  16.9% heart failure (10/59)  1.7% coronary insufficiency (1/59)  1.7% hypocholesterolemia (1/59)  1.7% coronary heart disease (1/59) | 100% (n= 63)  96.8% hypertension (61/63)  4.8% cardiovascular prophylaxis (3/63)  1.6% cardiac insufficiency (1/63)  1.6% glaucoma (1/63)  1.6% myocard infarction (1/63) | 74.2% (n= 66)  48.5% hypertension (32/66)  47.0% heart failure (31/ 66)  3.0% coronary insufficiency (2/66) |
| **Dose of reported suspected ACEi/ARB** | | | | |
| **Information reported**  **Normal**  **Increased**  **Decreased** | 100.0% (n= 51)  96.1% (49/51)  2.0% (1/51)  2.0% (1/51) | 63.0% (n= 51)  90.2% (46/51)  3.9% (2/51)  5.9% (3/51) | 98.4% (n= 62)  90.3% (56/62)  6.5% (4/62)  3.2% (2/62) | 69.7% (n= 62)  95.2% (59/62)  0.0% (0/62)  4.8% (3/62) |
| **Treatment duration of ACEi/ARB until angioedema occurrence** | | | | |
| **Information reported**  **1-3 days**  **4-14 days**  **> 14 days – 2months**  **> 2 months – 1 year**  **> 1 year**  **Mean number of days (+/-SD)**  **Median number of days [IQR]** | 94.1% (n= 48)  4.2% (2/48)  2.1% (1/48)  6.3% (3/48)  8.3% (4/48)  79.2% (38/48)  NA  NA | 53.1% (n= 43)  27.9% (12/43)  25.6% (11/43)  18.6% (8/43)  11.6% (5/43)  16.3% (7/43)  224.7 (+/- 512.0)  14 [2.5-119.0] | 96.8% (61/63)  3.3% (2/61)  6.6% (4/61)  1.6% (1/61)  19.7% (1/61)  68.9% (42/61)  NA  NA | 64.0% n= 57)  28.1% (16/57)  19.3% (11/57)  8.8% (5/57)  19.3% (11/57)  24.6% (14/57)  678.3 (+/- 1347.3)  34 [3.0-365.0] |
| **Duration of angioedema occurrence after exposure** | | | | |
| **Information reported**  **< 1 hour**  **1-12 hours**  **> 12 hours** | 96.1% (n= 49)  8.2% (4/19)  63.3% (31/49)  28.6% (14/49) | 1.2% (n= 1)  100% (1/1)  0.0% (0/1)  0.0% (0/1) | 95.2% (n= 60)  5.0% (3/60)  50.0% (30/60)  45.0% (27/60) | 5.6% (n= 5)  40.0% (2/5)  60.0% (3/5)  0.0% (0/5) |
| **Action taken with ACEi/ARB** | | | | |
| **Information reported**  **Withdrawal**  **Dose reduced**  **Drug not withdrawn** | 100% (n= 51)  62.7% (32/51)  0.0% (0/51)  37.3% (19/51) | 81.5% (n= 66)  90.9% (60/66)  0.0% (0/66)  9.1% (6/66) | 98.4% (n= 62)  77.4% (48/62)  0.0% (0/62)  22.6% (14/62) | 77.5% (n= 69)  92.8% (64/69)  1.4% (1/69)  5.8% (4/69) |
| **Re-exposure with the respective ACEi/ARB** | | | | |
| **Information reported**  **Re-exposure**  **Angioedema after re-exposure**  **Yes**  **No**  **Unknown** | 88.2% (n= 45)  33.3% (15/45)  60.0% (9/15)  20.0% (3/15)  20.0% (3/15) | NA  NA  NA NA NA | 73.0% (n= 46)  37.0% (17/46)  70.6% (12/17)  29.4% (5/17)  0.0% (0/17) | NA  NA  NA  NA  NA |

BMI= body mass index; SD= standard deviation; IQR= interquartile range; NA= information was not available in the respective dataset; ACEi= angiotensin converting enzyme inhibitors; ARB= angiotensin receptor blocker

Appendix 3 Table 2 shows the descriptive analyses of ACEi/ARB therapy in reports referring to females and males from the vARIANCE study and in EudraVigilance reports.

Differences were observed for females and males in EudraVigilance reports. Sacubitril/valsartan was in proportion more frequently reported in angioedema reports referring to males than to females. Coronary artery diseases were in proportion more frequently reported as indication of ACEi/ARB therapy in angioedema reports referring to males than to females.

Appendix 3 Table 3) Analyses of angioedema reported in EudraVigilance and the vARIANCE study.

|  | **Females: Angioedema cases vARIANCE study (n= 51)** | **Females: Angioedema reports EudraVigilance (n= 81)** | **Males: Angioedema cases vARIANCE study (n= 63)** | **Males: Angioedema reports EudraVigilance (n= 89)** |
| --- | --- | --- | --- | --- |
| **The five most frequently reported locations of angiodema** | | | | |
| **Information reported**  **1.**  **2.**  **3.**  **4.**  **5.** | 100.0% (n= 51)  66.7% lips (34/51)  54.9% face (28/51)  49.0% tongue (25/51)  33.3% eye lid (17/51)  27.5% oral mucosa (14/51) | 100.0% (n= 81)  32.1% tongue (26/81)  29.6% face (24/81)  25.9% lips (21/81)  7.4% blepharon (6/81)  7.4% eyes (6/81)  6.2% pharynx (5/81)  6.2% throat (5/81) | 100.0% (n= 63)  74.6% lips (47/63)  54.0% face (34/63)  42.9% tongue (27/63)  30.2% throat (19/63)  19.0% oral mucosa (12/63) | 100.0% (n= 89)  24.7% lips (22/89)  24.7% tongue (22/89)  22.5% face (20/89)  7.9% eyes (7/89)  7.9% larynx (7/89) |
| **Duration of angioedema** | | | | |
| **Information reported**  **< 1 day**  **1-3 days**  **> 3 days**  **Mean number of days (+/-SD)**  **Median number of days [IQR]** | 98.0% (n= 50)  40.0% (20/50)  40.0% (20/50)  20.0% (10/50)  -  - | 29.6% (n= 24)  12.5% (3/24)  41.7% (10/24)  45.8% (11/24)  9.9 (+/-24.2)  2.5 [1.8-6.0] | 100.0% (n= 63)  33.3% (21/63)  52.4% (22/63)  14.3% (9/63)  -  - | 20.2% (n= 18)  16.7% (3/18)  50.0% (9/18)  33.3% (6/18)  5.8 (+/-9.4)  3 [1.3-4.8] |
| **Analysis of associated factors** | | | | |
| **Associated factors reported**  **The three most frequently reported associated factors** | 19.6% (n= 10)  50.0% infection (5/10)  30.0%% stress (3/10)  20.0% operation (2/10) | 25.9% (n= 21)  28.6% product substitution issue (6/21)  14.3% everolimus (3/21)  14.3% infection (3/21) | 11.1% (n= 7)    28.6% stress (2/7)  28.6% infection (2/7) | 23.6% (n= 21)    14.3% product substitution issue (3/21)  14.3% vaccination (3/21)  9.5% apixaban (2/21) |
| **Corrective treatment of angioedema** | | | | |
| **Corrective treatment of angioedema received**  **Antihistamines**  **Cortisone**  **Epinephrine**  **C1-esterase inhibitors**  **Icatibant**  **Response to corrective treatment reported**  **Immediately**  **< 6 hours** | 86.3% (n= 44)  63.6% (28/44)  86.4% (38/44)  0.0% (0/44)  0.0% (0/44)  0.0% (0/44)  59.1% (26/44)  -  - | 25.9% (n= 21)  100.0% (21/21)  76.2% (16/21)  14.3% (3/21)  0.0% (0/21)  9.5% (2/21)  23.8% (5/21)  20.0% (1/5)  40.0% (2/5) | 82.3% (n= 51)  56.9% (29/51)  72.5% (37/51)  2.0% (1/51)  0.0% (0/51)  0.0% (0/51)  52.9% (27/51)  -  - | 33.7% (n= 30)  73.3% (22/30)  80.0% (24/30)  20.0% (6/30)  13.3% (4/30)  3.3% (1/30)  26.7% (8/30)  12.5% (1/8)  25.0% (2/8) |

Appendix 3 Table 3 shows the descriptive analyses of angioedema in females and males in cases from the vARIANCE study and in EudraVigilance reports.

Differences between sexes were observed with regard to the location of angioedema in both datasets. However, the results between both data sets were inconsistent. Thus, no sex-differences regarding the locations of angioedema are assumed.
