# Supplementary material for: Non‐genetic factors associated with ACE‐inhibitor and angiotensin receptor blocker‐induced angioedema
Source: Clin Transl Allergy. 2025 May 7;15(5):e70058. doi: 10.1002/clt2.70058 (PMC12058302; doi:10.1002/clt2.70058)
Supplement: Supplementary file 4 — Supporting Information S4 [file CLT2-15-e70058-s002.docx]

**Appendix 4) ACEi/ARB-stratified descriptive analyses of reports and cases from EudraVigilance and the vARIANCE study.**

Appendix 4 Table 1) Analyses of data of the patients included in angioedema reports and cases from EudraVigilance and the vARIANCE study.

|  | **ACEi angioedema cases vARIANCE study (n= 96)*** | **ACEi angioedema reports EudraVigilance (n= 69)** | **ARB angioedema cases vARIANCE study (n= 13)*** | **ARB angioedema reports EudraVigilance (n= 102)** |
| --- | --- | --- | --- | --- |
| **Demographical parameters of the patients** | |  |  |  |
| **Age**  **Information reported**  **Mean**  **Median**  **Sex**  **Female**  **Male**  **Unknown**  **BMI**  **Information reported**  **Mean**  **Median**  **Ethnicity**  **Information reported**  **European/Caucasian**  **Asian**  **African**  **Others** | 99.0% (n= 95)  64.4 (+/-11.0)  64.0 [55.0-72.0]  43.8% (n= 42)  56.3% (n= 54)  0.0% (n= 0)  97.9% (n= 94)  29.1 (+/-5.9)  28.3 [25.0-32.0]  96.9% (n= 93)  92.5% (86/93)  3.2% (3/93)  2.2% (2/93)  2.2% (2/93) | 89.9% (n= 62)  66.5 (+/-14.5)  70.0 [58.5-77.0]  47.4% (n= 81)  52.0% (n= 89)  0.0% (n= 0)  49.3% (n= 34)  27.5 (+/-4.4)  26.5 [24.6-29.7]  NA  NA  NA  NA  NA | 84.6% (n= 11)  67.9 (+/-11.4)  69.0 [60.0-76.0]  53.8% (n= 7)  46.2% (n= 6)  0% (n= 0)  92.3% (n= 12)  28.5 (+/-3.2)  28.4 [26.3-29.8]  92.3% (n= 12)  100.0% (12/12)  0.0% (0/12)  0.0% (0/12)  0.0% (0/12) | 67.6% (n= 69)  68.4 (+/-13)  70.0 [58.0-80.0]  48.0% (n= 49)  51.0% (n= 52)  1.0% (n= 0)  30.4% (n= 31)  29.4 (+/-6.3)  28.7 [25.0-33.3]  NA  NA  NA  NA  NA |
| **Lifestyle factors of the patients** | |  |  |  |
| **Alcohol consumption**  **Current smoker**  **Former smoker** | 50.0% (n= 48)  17.7%% (n= 17)  47.9% (n= 46) | 4.3% (n= 3)  7.2% (n= 5)  0.0% (n= 0) | 69.2% (n= 9)  15.4% (n= 2)  53.8% (n= 7) | 3.9% (n= 4)  2.0% (n= 2)  1.0% (n= 1) |
| **Allergies^1^** |  |  |  |  |
| **Allergies reported**  **Intolerances reported**  **Most frequently reported allergies/intolerances** | 32.3% (n= 31)  48.4% pollen/dustmite  (15/31)  25.8% drugs  (8/31)  19.4% food  (6/31) | 13.0% (n= 9)  1.4% (n= 1)  60.0% drugs (6/10)  50.0% pollen/dustmite(5/10) | 38.5% (n= 5)  60.0% drugs (3/5)  15.4% pollen/dustmite (2/5)  15.4% others (2/5) | 7.8% (n= 8)  2.9% (n= 3)  40.0% drugs (4/10)  30.0% food (3/10)  20.0% nickel (2/10)  20.0% pollen/dustmite (2/10) |
| **Previous swellings** | |  |  |  |
| **Previous swellings reported**  **Number of previous swellings**  **Once**  **2-5 times**  **6-10 times**  **> 10 times**  **Previous swelling related to drugs**  **Previous swellings related to ACEi/ARBs**  **Previous swellings related to other causes**  **Most frequently reported other causes**  **Food**  **Operation**  **Stress**  **Infection**  **Physical activity**  **Temperature (heat, cold)** | 56.3% (n= 54)  20.4% (11/54)  46.3% (25/54)  16.7% (9/54)  14.8% (8/54)  70.4% (38/54)  62.9% (34/54)  11.1% (6/54)  83.3% (5/6)  16.7% (1/6)  16.7% (1/6)  0.0% (0/6)  0.0% (0/6)  0.0% (0/6) | 18.8% (n= 13)  NA  NA  NA  NA  NA  76.9% (10/13)  NA  NA  NA  NA  NA  NA  NA | 61.5% (n= 8)  0.0% (0/8)  50.0% (4/8)  12.5% (1/8)  12.5% (1/8)  75.0% (6/8)  75.0% (6/8)  50.0% (4/8)  25.0% (1/4)  25.0% (1/4)  0.0% (0/4)  25.0% (1/4)  25.0% (1/4)  25.0% (1/4) | 2.0% (n= 2)  NA  NA  NA  NA  NA  50.0% (1/2)  NA  NA  NA  NA  NA  NA  NA |
| **Histories of the patients^2^** | | |  |  |
| **Histories reported**  **The three most frequently reported patient histories** | 80.2% (n= 77)  87.0% hypertension  (67/77)  33.8% type 2 diabetes mellitus (20/77)  19.5% hypothyroidism  (15/77) | 81.2% (n= 56)  55.4% hypertension (31/56)  12.5% type 2 diabetes mellitus (7/56)  7.1% coronary artery disease (4/56)  7.1% drug hypersensitivity (4/56)  7.1% essential hypertension (4/56)  7.1% gastroesophageal reflux disease (4/56)  7.1% hyperlipidemia (4/56)  7.1% seasonal allergy (4/56) | 76.9% (n= 10)  90.0% hypertension (9/10)  20.0%% hypothyroidism (2/10)  20.0% atrial fibrillation (2/10) | 53.9% (n= 55)  47.3% hypertension (26/55)  14.5% coronary artery disease (8/55)  12.7% obesity (7/55) |
| **Seriousness of angioedema reports/cases^3^** | | | | |
| **Serious**  **Death**  **Life-threatening**  **Hospitalisation**  **Disabling**  **Ambulance** | NA  NA  26.0% (n= 25)  52.1% (n= 51)  NA  44.8% (n= 43) | 84.1% (n= 58)  4.3% (n= 3)  20.3% (n= 14)  50.7% (n= 35)  0.0% (n= 0)  NA | NA  NA  30.8% (n= 4)  46.2% (n= 6)  NA  46.2% (n= 6) | 55.9% (n= 57)  2.0% (n= 2)  3.9% (n= 4)  17.6% (n= 18)  1.0% (n= 1)  NA |

BMI= body mass index; SD= standard deviation; IQR= interquartile range; NA= information was not available in the respective data set; ACEi= angiotensin converting enzyme inhibitors; ARB= angiotensin receptor blocker

*in 5 cases of the vARIANCE study an ACEi and an ARB was reported as suspected, these cases were therefore not assigned to either of the groups.

^1^ more than one allergy or intolerance can be reported per patient.

^2^ in the vARIANCE study some diseases were specifically queried in the questionnaire. In EudraVigilance, no specific diseases are queried. The reported histories in EudraVigilance were analysed on the PT-level of MedDRA terminology.

^3^ in the vARIANCE study the reporter could specify whether the respective angioedema was life-threatening, led to hospitalization or a visit of an ambulance. In EudraVigilance, the classification of seriousness of reports follows the legal definition of seriousness. In EudraVigilance a report is classified as serious if the reported ADR was life-threatening, led to death, hospitalisation or prolongation thereof, congenital anomalies or permanent disability.

Appendix 4 Table 1 shows the descriptive analyses of the patients populations included in angioedema cases related to ACEi and ARBs from the vARIANCE study and angioedema reports from EudraVigilance.

Differences between angioedema reports related to ACEi and ARBs were observed with regard to histories of the patients (only EudraVigilance) and the seriousness of angioedema reports (only EudraVigilance). Coronary artery diseases were in proportion more frequently reported for patients with ARB- than for ACEi-associated angioedema in EudraVigilance reports. In EudraVigilance reports, reports of ARB-associated angioedema were in proportion less frequently classified as serious than reports of ACEi-associated angioedema.

Appendix 4 Table 2) Analyses of data concerning ACEi/ARB therapy in angioedema reports and cases from EudraVigilance and the vARIANCE study.

|  | **ACEi angioedema cases vARIANCE study (n= 96)** | **ACEi angioedema reports EudraVigilance (n= 69)** | **ARB angioedema cases vARIANCE (n= 13)** | **ARB angioedema reports EudraVigilance (n= 102)** |
| --- | --- | --- | --- | --- |
| **The five most frequently suspected ACEi/ARBs** | | | | |
| **Information reported**  **1.**  **2.**  **3.**  **4.**  **5.** | 99.0% (n= 95)  68.4% ramipril (65/95)  15.6% lisinopril (15/95)  10.5% enalapril (10/95)  2.1% lisinopril/HCT (2/95)  2.1% perindopril (2/95) | 100.0% (n= 69)  68.1% ramipril (47/69)  8.7% enalapril (6/69)  7.2% Lisinopril (5/69)  5.8% Ramipril/HCT (4/69)  2.1% perindopril (2/69) | 100.0% (n= 13)  53.8% candesartan (7/13)  15.4% valsartan (2/13)  7.7% candesartan/HCT (1/13)  7.7% irbesartan (1/13)  7.7% losartan (1/13)  7.7% sacubitril/valsartan (1/13)  7.7% telmisartan (1/13) | 100.0% (n= 102)  50.0% sacubitril/valsartan (51/102)  29.4% candesartan (30/102)  7.8% valsartan (8/102)  2.9% candesartan/HCT (3/102)  2.9% valsartan/HCT (3/102) |
| **Indication of ACEi/ARB therapy** | | | | |
| **Information reported**  **1.**  **2.**  **3.** | 100% (n= 114)  96.9% hyptension (93/114)  1.0% cardiac insufficiency (1/114)  1.0% cardiovascular prophylaxis (1/114)  1.0% glaucoma (1/114)  1.0% myocard infarct (1/114) | 72.5% (n= 50)  90.0% hypertension (45/50) | 100% (n= 13)  92.3% hypertension (12/13)  7.7% cardiac insufficiency (1/13) | 74.5% (n= 76)  51.3% heart failure (39/76)  47.4 % hypertension (36/76) |
| **Dose of reported suspected ACEi/ARB** | | | | |
| **Information reported**  **Normal**  **Increased**  **Decreased** | 100.0% (n= 96)  93.8% (90/96)  3.1% (3/96)  3.1% (3/96) | 62.3% (n= 43)  100.0% (43/43)  0.0% (0/43)  0.0% (0/43) | 92.3% (n= 12)  100.0% (12/12)  0.0% (0/12)  0.0% (0/12) | 69.6% (n= 71)  88.7% (63/71)  2.8% (2/71)  8.5% (6/71) |
| **Treatment duration of ACEi/ARB until angioedema occurrence** | | | | |
| **Information reported**  **1-3 days**  **4-14 days**  **> 14 days – 2months**  **> 2 months – 1 year**  **> 1 year**  **Mean number of days (+/-SD)**  **Median number of days [IQR]** | 94.8% (n= 91)  4.4% (4/91)  5.5% (5/91)  4.4% (4/91)  14.3% (13/91)  71.4% (65/91)  NA  NA | 56.5% (n= 39)  17.9% (7/39)  20.5% (8/39)  7.7% (3/39)  17.9% (7/39)  35.9% (14/39)  950.7 (+/- 1,545.2)  120.0 [7.0-860.0] | 100% (n= 13)  0.0% (0/13)  0.0% (0/13)  0.0% (0/13)  7.7% (1/13)  92.3% (12/13)  NA  NA | 60.8% n= 62)  33.9% (21/62)  24.2% (15/62)  16.1% (10/62)  14.5% (9/62)  11.3% (7/62)  181.5 (+/- 462.4)  10.5 [2.0-66.3] |
| **Duration of angioedema occurrence after exposure** | | | | |
| **Information reported**  **< 1 hour**  **1-12 hours**  **> 12 hours** | 95.8% (n= 92)  7.6% (7/92)  54.3% (50/92)  38.0% (35/92) | 5.8% (n= 4)  50.0% (2/4)  50.0% (2/4)  0.0% (0/4) | 100.0% (n= 13)  0.0% (0/13)  69.2% (9/13)  30.8% (4/13) | 2.0% (n= 2)  50.0% (1/2)  50.0% (1/2)  0.0% (n= 0) |
| **Action taken with ACEi/ARB** | | | | |
| **Information reported**  **Withdrawal**  **Dose reduced**  **Drug not withdrawn** | 99.0% (n= 95)  69.5% (66/95)  0.0% (0/95)  30.5% (29/95) | 71.0% (n= 49)  93.6% (46/49)  0.0% (0/49)  6.1% (3/49) | 100.0% (n= 13)  69.2% (9/13)  0.0% (0/13)  30.8% (4/13) | 85.3% (n= 87)  90.8% (79/87)  1.1% (1/87)  8.0% (7/87) |
| **Re-exposure with the respective ACEi/ARB** | | | | |
| **Information reported**  **Re-exposure**  **Angioedema after re-exposure**  **Yes**  **No**  **Unknown** | 88.5% (n= 85)  32.9% (28/85)  64.3% (18/28)  25.0% (7/28)  10.7% (3/28) | NA  NA  NA  NA  NA | 84.6% (n= 11)  18.2% (2/11)  50.0% (1/2)  50.0% (1/2)  0.0% (0/2) | NA  NA  NA  NA  NA |

BMI= body mass index; SD= standard deviation; IQR= interquartile range; NA= information was not available in the respective data set; ACEi= angiotensin converting enzyme inhibitors; ARB= angiotensin receptor blocker

Appendix 4 Table 2 shows the descriptive analyses of ACEi/ARB therapy in cases of the vARIANCE study and reports from EudraVigilance.

Differences were observed for ACEi and ARBs with regard to the indication of reported ACEi/ARB therapy in EudraVigilance reports. Coronary artery diseases were in proportion more frequently reported as indication of ARB than ACEi therapy in EudraVigilance reports.

Appendix 4 Table 3) Analyses of angioedema reported in EudraVigilance and the vARIANCE study.

|  | **ACEi angioedema cases vARIANCE study (n= 96)** | **ACEi angioedema reports EudraVigilance (n= 69)** | **ARB angioedema cases vARIANCE (n= 13)** | **ARB angioedema reports EudraVigilance (n= 102)** |
| --- | --- | --- | --- | --- |
| **The five most frequently reported locations of swellings** | |  |  |  |
| **Information reported**  **1.**  **2.**  **3.**  **4.**  **5.** | 100% (n= 96)  69.8% lips (67/96)  57.3% face (55/96)  43.8% tongue (42/96)  28.1% throat (27/96)  22.9% oral mucosa (22/96) | 100.0% (n= 69)  37.7% tongue (26/69)  31.9% lip (22/69)  17.4% face (12/69)  8.7% throat (6/69)  5.8% larynx (4/69)  5.8% eyes (4/69) | 100.0% (n= 13)  69.2% lips (9/13)  61.5% tongue (8/13)  46.2% pharynx (6/13)  38.5% face (5/13)  38.5% eye lid (5/13) | 100.0% (n= 102)  31.4% face (32/102)  21.6% tongue (22/102)  20.6% lips (21/102)  8.8% eyes (9/102)  7.8% pharynx (8/102) |
| **Duration of swelling** |  |  |  |  |
| **Information reported**  **< 1 day**  **1-3 days**  **> 3 days**  **Mean number of days (+/-SD)**  **Median number of days [IQR]** | 100.0% (n= 69)  38.5% (37/69)  43.8% (42/69)  17.7% (17/69)  NA  NA | 27.5% (n= 19)  15.8% (3/19)  47.4% (9/19)  36.8% (7/19)  5.1 (+/-7.3)  2.0 [1.5-4.5] | 92.3% (n= 12)  16.7% (2/12)  75.0% (9/12)  8.3% (1/12)  NA  NA | 22.5 (n= 23)  13.0% (3/23)  43.5% (10/23)  43.5% (10/23)  10.7 (+/-25.1)  3 [1.5-5.5] |
| **Analysis of associated factors** | | |  |  |
| **Associated factors reported**  **Most frequently reported associated factors** | 13.5% (n= 13)  46.2% infection (6/13)  23.1%% stress (3/13) | 26.1% (n= 18)  16.7% everolimus (3/18)  11.1% apixaban (2/18)  11.1% infection (2/18)  11.1% racecadotril (2/18)  11.1% simvastatin (2/18)  11.1% vaccination (2/18) | 23.1% (n= 3)  66.6% stress (2/3)  33.3% infection (1/3) | 24.5% (n= 25)  36.0% product substitution issue (9/25)  8.0% infection (2/25)  8.0% operation (2/25)  8.0% unapproved splitting of tablets (2/25) |
| **Corrective treatment of swellings** |  |  |  |  |
| **Corrective treatment of swellings received**  **Antihistamines**  **Cortisone**  **Epinephrine**  **C1-esterase inhibitors**  **Icatibant**  **Response to corrective treatment reported**  **Immediately**  **< 6 hours** | 84.4% (n= 81)  60.5% (49/81)  80.2% (65/81)  1.2% (1/81)  0.0% (0/81)  0.0% (0/81)  54.3% (44/81)  NA  NA | 53.6% (n= 37)  91.9% (34/37)  81.1% (30/37)  18.9% (7/37)  10.8% (4/37)  5.4% (2/37)  27.0% (10/37)  20.0% (2/10)  40.0% (4/13) | 69.2% (n= 9)  55.6% (5/9)  66.7% (6/9)  0.0% (0/9)  0.0% (0/9)  0.0% (0/9)  66.7% (6/9)  NA  NA | 13.7% (n= 14)  64.3% (9/14)  71.4% (10/14)  14.3% (2/14)  7.1% (1/14)  0.0% (0/14)  21.4% (3/14)  0.0% (0/3)  0.0% (0/3) |

Appendix 4 Table 3 shows the analyses of angioedema reported in EudraVigilance and the vARIANCE study.

Differences between ACEi and ARBs were observed with regard to the location of angioedema in both data sets. However, the results between both data sets were inconsistent. Thus, no differences regarding the locations of angioedema are assumed.
