# Supplementary material for: Non‐genetic factors associated with ACE‐inhibitor and angiotensin receptor blocker‐induced angioedema
Source: Clin Transl Allergy. 2025 May 7;15(5):e70058. doi: 10.1002/clt2.70058 (PMC12058302; doi:10.1002/clt2.70058)
Supplement: Supplementary file 5 — Supporting Information S5 [file CLT2-15-e70058-s005.docx]

**Appendix 5) Descriptive analyses of reference reports of other ADRs to ACEi/ARB in EudraVigilance.**

Appendix 5 Table 1) Descriptive analyses of reference reports of other ADRs to ACEi/ARB in EudraVigilance.

| **EudraVigilance** | **ACEi/ARBs: Angioedema reports (n= 171)^1^** | **ACEi/ARBs: Reference reports (n= 4,650)^2^** |
| --- | --- | --- |
| **Demographical parameters** | | |
| **Age**  **Mean (+/-sd)**  **Median [IQR]**  **Unknown**  **Sex**  **Female**  **Male**  **Unknown**  **BMI**  **Mean (+/-sd)**  **Median [IQR]**  **Unknown** | 76.6% (n= 131)  67.5 (+/-14.2)  70.0 [58.0-78.5]  47.4% (n= 81)  52.0% (n= 89)  0.6% (n= 1)  38.0% (n= 65)  28.4 (+/-5.4)  26.6 [24.7-30.9] | 63.5% (n= 2,968)  69.2 (+/-13.5)  71.0 [61.0-79.0]  46.3% (n= 2.,41)  51.1% (n= 2,363)  3.4% (n= 158)  30.0% (n= 1,395)  28.1 (+/-5.6)  27.1 [24.3-31.1] |
| **Lifestyle factors of the patients^3^** |  |  |
| **Alcohol consumption**  **Current smoker**  **Former smoker** | 4.1% (n= 7)  4.1% (n= 7)  0.6% (n= 1) | 1.0% (n= 46)  1.7% (n= 81)  0.0% (n= 0) |
| **Allergies and intolerances^3^** |  |  |
| **Allergy**  **Intolerances**  **Summarized** | 9.9% (n= 17)  2.3% (n= 4)  12.3% (n= 21) | 3.0% (n= 139)  0.9% (n= 43)  3.7% (n= 173) |
| **History of angioedema^3^** |  |  |
| **History of angioedema reported**  **Prior angioedema associated with ACEi/ARB** | 8.8% (n= 15)  73.3/ (11/15) | 0.0% (n= 0)  0.0% (0/0) |
| **The five most frequently reported histories (PT-level) of the patients^4^** | | |
| **History reported**  **1.**  **2.**  **3.** | 64.9% (n= 111)  51.4% hypertension (57/111)  11.7% type 2 diabetes mellitus (13/111)  10.8% coronary artery disease (12/111) | 44.8% (n= 2,082)  46.0% hypertension (958/2,082)  8.8% atrial fibrillation (183/2,082)  8.4% coronary artery disease (174/2,082) |
| **Seriousness criteria of the ADR reports^5^** | | |
| **Serious**  **Death**  **Life-threatening**  **Hospitalisation**  **Disabling** | 67.3% (n= 115)  2.9% (n= 5)  10.5% (n= 18)  31.0% (n= 53)  6.6% (n= 1) | 44.4% (n= 2,054)  2.3% (n= 106)  3.8% (n= 174)  27.0% (n= 1.247)  0.8% (n= 35) |
| **Most five most frequently reported ACEi/ARBs reported as suspected/interacting^6^** | | |
| **Information reported**  **1.**  **2.**  **3.**  **4.**  **5.** | 100.0% (n= 171)  29.8% sacubitril/valsartan (51/171)  27.5% ramipril (47/171)  17.5% candesartan (30/171)  4.7% valsartan (8/171)  3.5% enalapril (6/171) | 100.0% (n= 4,650)  30.1% sacubitril/valsartan (1,398/4,650)  17.9% ramipril (831/4,650)  17.8% candesartan (828/4,650)  8.5% valsartan (397/4,650)  3.7% candesartan/HCT (171/4,650) |
| **The five most frequently reported ADRs (PT-level)^7^** | | |
| **Information reported**  **1.**  **2.**  **3.**  **4.**  **5.** | 100.0% (n= 171)  44.4% angioedema (76/171)  27.5% swollen tongue (47/171)  19.9% swelling face (34/171)  18.7% lip swelling (32/171)  12.3% dyspnea (21/171) | 100.0% (n= 4,650)  10.2% dizziness (472/4,650)  6.9% nausea (320/4,650)  6.6% hypotension (305/4,650)  6.4% cough (298/4,650)  5.4% pruritus (251/4,650) |

^1^ all ADR reports with an at least possible causal relationship between the intake of the suspected/interacting ACEi/ARB and the occurrence of an angioedema.

^2^ all ADR reports including ACEi/ARBs as suspected/interacting drugs excluding reports for patients < 16 years, reports related to drug exposure during pregnancy and reports coded with an ADR related to the SMQ “angioedema (narrow)”.

^3^ Lifestyle factors, allergies and intolerances as well as a history of previous angioedema were analysed based on appropriate Terms of MedDRA terminology. Note that, this information may have been reported in the free-text information and, thus, may also been collected during the manual assessment of the angioedema reports with an at least possible causal relationship, but not in the reference reports.

^4^ more than one history of the patient can be reported per ADR report. The histories of the patients were analysed based on the PT-level of MedDRA terminology.

^5^ the definition of the seriousness criteria follows the legal definition of seriousness of the AMG. The seriousness may not correspond to the clinical severity of an ADR. More than one seriousness criterion can be reported per ADR report.

^6^ more than one ACEi/ARB can be reported as suspected/interacting per ADR-report.

^7^ more than one ADR can be reported per ADR report. The ADRs were analysed based on the PT-level of MedDRA terminology.
