# Supplementary material for: Non‐genetic factors associated with ACE‐inhibitor and angiotensin receptor blocker‐induced angioedema
Source: Clin Transl Allergy. 2025 May 7;15(5):e70058. doi: 10.1002/clt2.70058 (PMC12058302; doi:10.1002/clt2.70058)
Supplement: Supplementary file 6 — Supporting Information S6 [file CLT2-15-e70058-s001.docx]

**Appendix 6 Figures of odds ratio calculations**

Appendix 6 Figure 1) Concomitant drugs reported more frequently in angioedema versus reference reports.


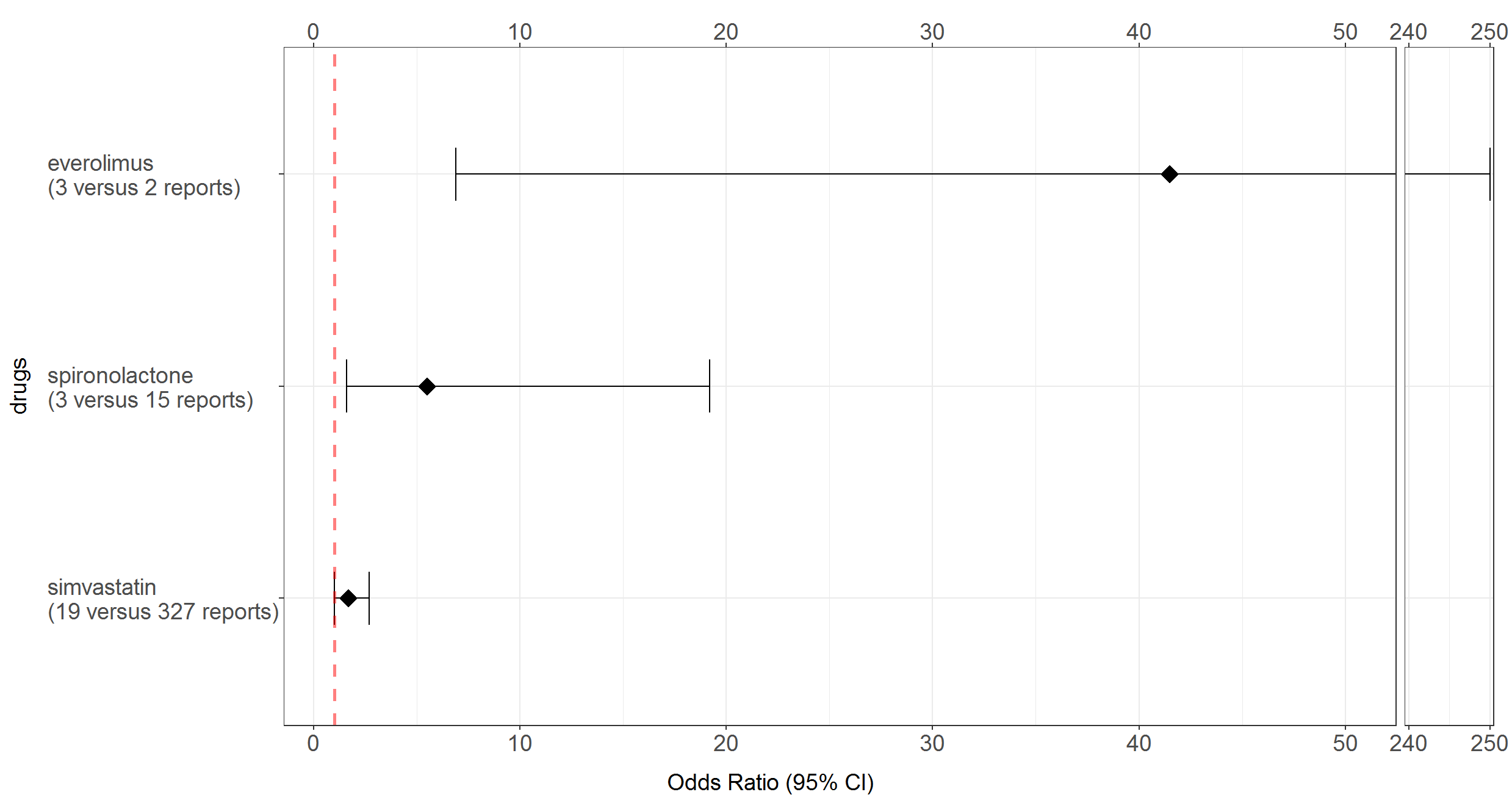


Appendix 6 Figure 1 shows the concomitant drugs more frequently reported in ACEi-/ARB-induced angioedema compared to ACEi/ARB reference reports. Additionally, the number of angioedema versus reference reports is displayed as absolute numbers. The calculation of ORs is based on two-by-two tables. If the lower CI was higher than 1, the respective concomitant drug was assumed to be more frequently reported in angioedema reports. If the upper CI was lower than 1, the respective concomitant drug was assumed to be more frequently reported in reference reports. None of the concomitant drugs was found to be more commonly reported in reference reports.

Appendix 6 Figure 2) Medical histories of the patients more frequently reported in angioedema versus reference reports.


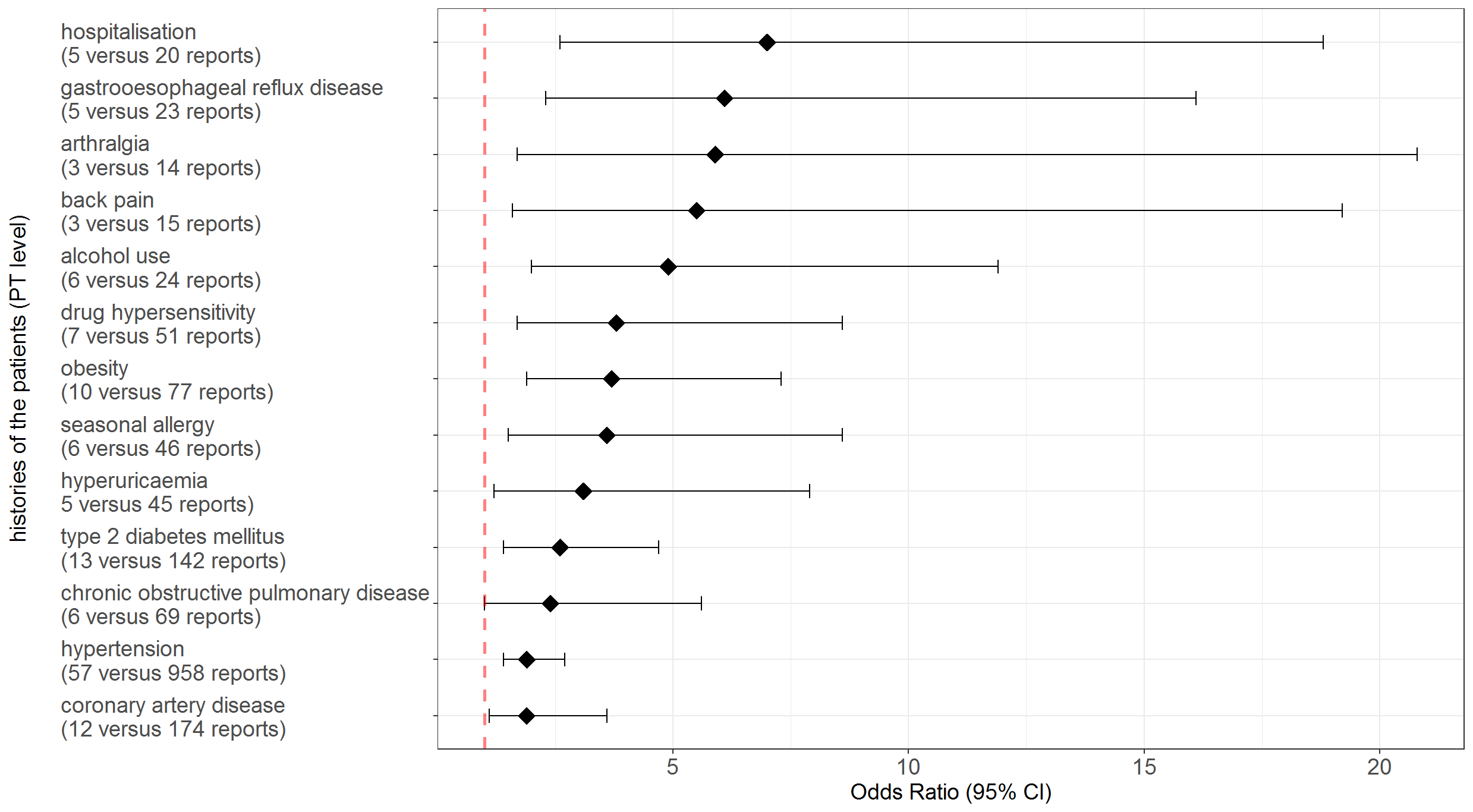


Appendix 6 Figure 2 shows the dedical histories of the patients more frequently reported in ACEi-/ARB-induced angioedema compared to ACEi/ARB reference reports. Additionally, the number of angioedema versus reference reports is displayed in absolute numbers. The calculation of ORs is based on two-by-two tables. If the lower CI was higher than 1, the respective patient’s history was assumed to be more frequently reported in angioedema reports. If the upper CI was lower than 1, the respective patient’s history was assumed to be more frequently reported in reference reports. None of the patient’s histories was found to be more commonly reported in reference reports. Notably, MedDRA terminology not only codes diseases but also conditions, laboratory results and investigations. In case of hospitalisation, this could present previous hospitalisations or the hospitalisation due to the reported angioedema.

Appendix 6 Figure 3) Co-reported ADRs more frequently reported in angioedema or reference reports.


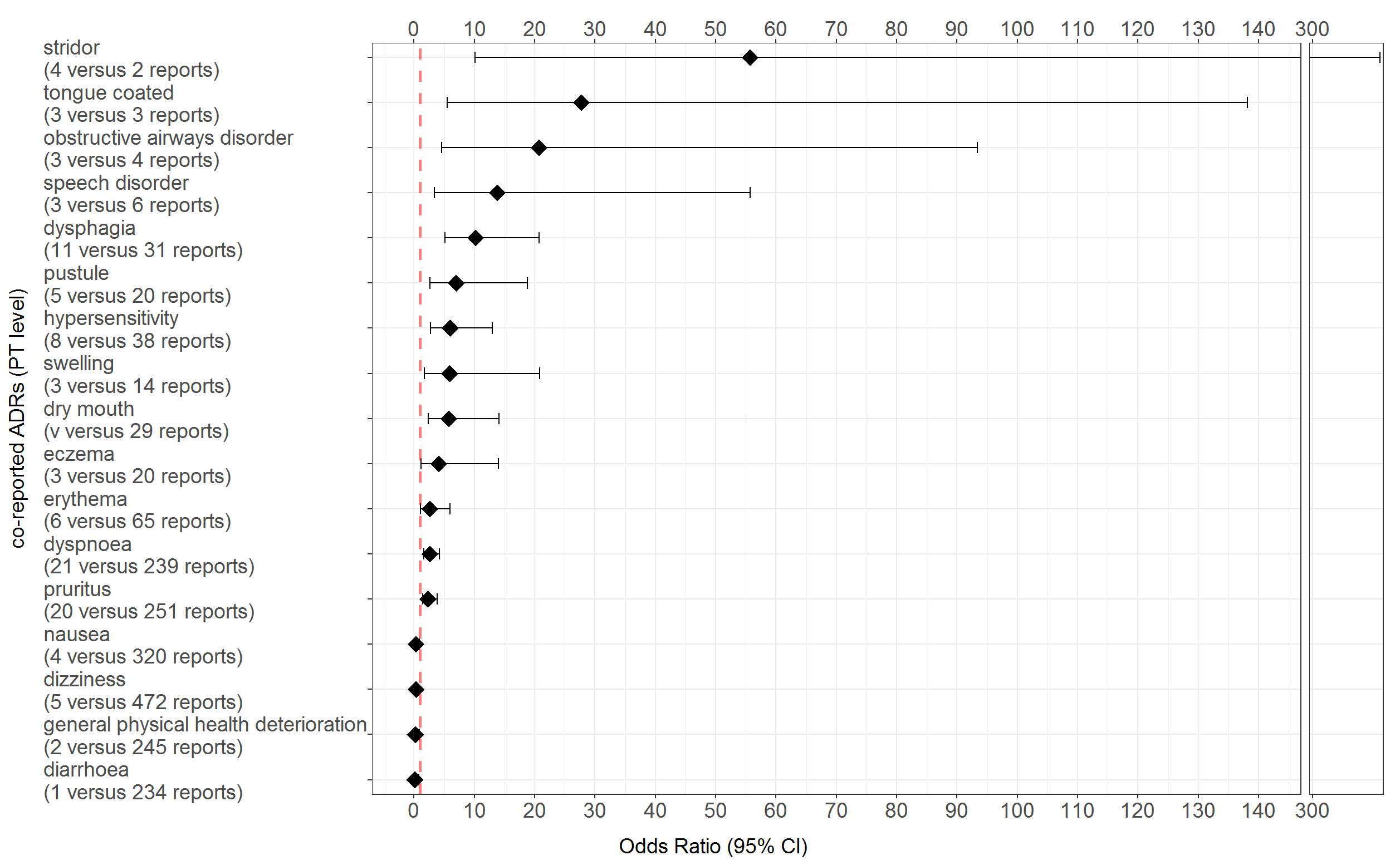


Appendix 6 Figure 3 shows the co-reported ADRs more frequently reported in ACEi-/ARB-induced angioedema compared to ACEi/ARB reference reports. Additionally, the number of angioedema versus reference reports is displayed in total counts. The calculation of ORs is based on two-by-two tables. If the lower CI was higher than 1, the respective co-reported ADR was assumed to be more frequently reported in angioedema reports. If the upper CI was lower than 1, the respective co-reported ADR was assumed to be more frequently reported in reference reports.
