# Supplementary material for: Non‐genetic factors associated with ACE‐inhibitor and angiotensin receptor blocker‐induced angioedema
Source: Clin Transl Allergy. 2025 May 7;15(5):e70058. doi: 10.1002/clt2.70058 (PMC12058302; doi:10.1002/clt2.70058)

**Appendix 7) Number of ACEi and ARB prescriptions in Germany.**

Appendix 7 Figure 1) Number of ACEi and ARB prescriptions per year in Germany.


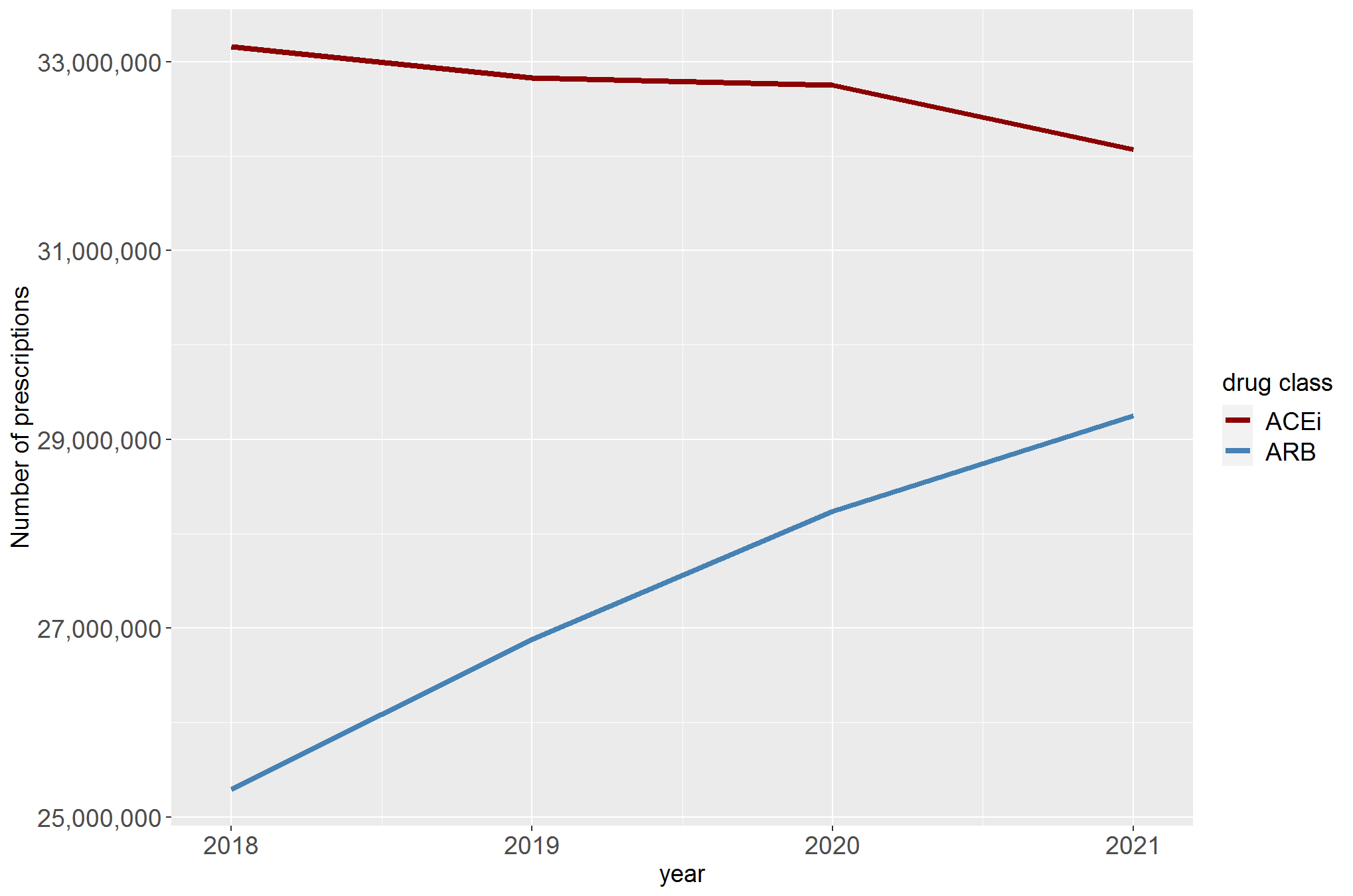


Appendix 7 Figure 2) Number of drug prescriptions per year for the individual ACEi in Germany.


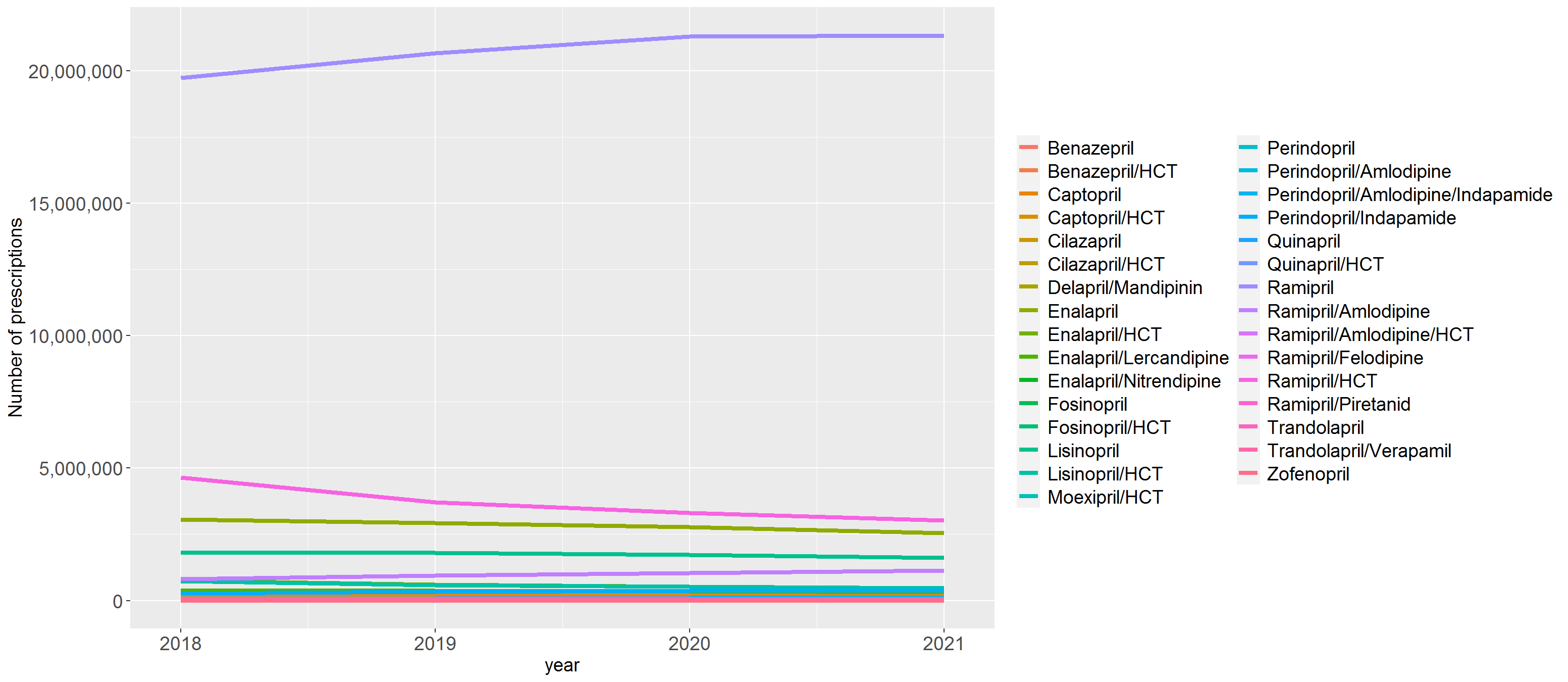


Appendix 7 Figure 3) Number of drug prescriptions per year for the individual ARB in Germany.


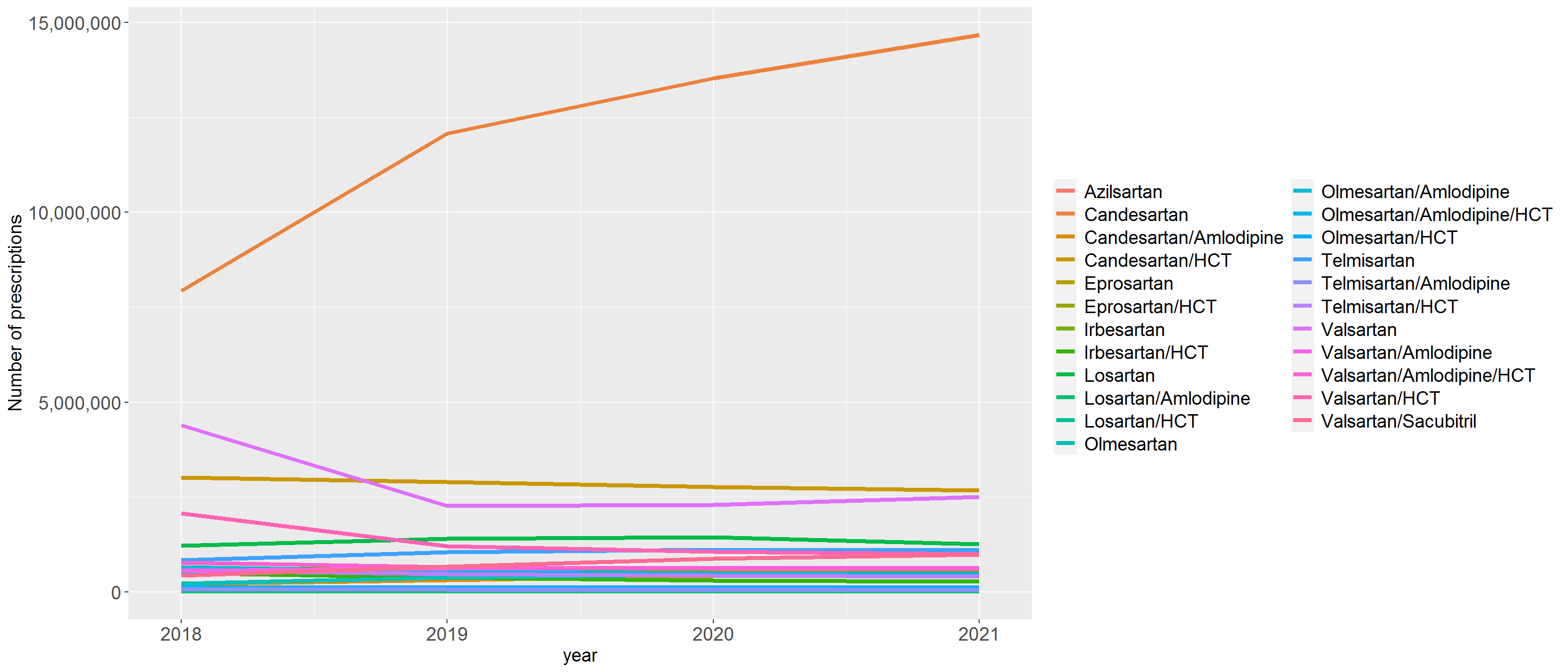

Supplement: Supplementary file 7 — Supporting Information S7 [file CLT2-15-e70058-s007.docx]
